# Enhancement of PID controller performance for a combined LFC and AVR single- and two-area model using exponential distribution optimization technique

**DOI:** 10.1038/s41598-025-29137-5

**Published:** 2025-12-23

**Authors:** Mohamed S. Amin, Mahmoud A. Attia, S. F. Mekhamer, Amr K. Khamees

**Affiliations:** 1 Department of Electrical Power and Machines, Faculty of Engineering, Ain Shams University, Cairo, Egypt; 2 Faculty of Engineering and Technology, Future University in Egypt, New Cairo, Egypt; 3 Department of Engineering Physical and Mathematics, Faculty of Engineering, Ain Shams University, Cairo, Egypt

**Keywords:** Proportional integral derivative controller, Automatic voltage regulator, Load frequency control, Exponential distribution optimization algorithm, Integral time square error, Integral time absolute error, Interconnected power system, Firefly algorithm, Nonlinear threshold-accepting algorithm, Electrical and electronic engineering, Energy grids and networks

## Abstract

This study presents the exponential distribution optimization (EDO) technique for tuning the proportional-integral-derivative (PID) controller parameters in combined load frequency control (LFC) and automatic voltage regulation (AVR) for both single- and two-area electrical power system models. The objective is to enhance system performance by minimizing frequency and voltage deviations. The proposed approach leverages widely adopted objective functions, namely, the integral time squared error (ITSE) and the integral time absolute error (ITAE) to effectively reduce control errors while improving system stability and transient response. The EDO algorithm iteratively refines PID parameters by selecting optimal values from evolving solution populations, with system disturbances introduced to evaluate robustness under varying operating conditions. Simulation results demonstrate significant improvements, including a 16.52% reduction in LFC undershoots and a 33.3% improvement in AVR steady-state error in single-area systems, and up to 13.2% faster settling times in two-area systems. The EDO-based controller achieves faster dynamic responses, smoother operation, and complete elimination of steady-state errors while maintaining balanced frequency overshoot and minimized transient oscillations. Comparative analyses confirm the method’s superiority over established optimization techniques. A comprehensive robustness further validates the reliability and effectiveness of the EDO-tuned PID controller, underscoring its strong potential for ensuring power system stability.

## Introduction

**Fig. 1:**
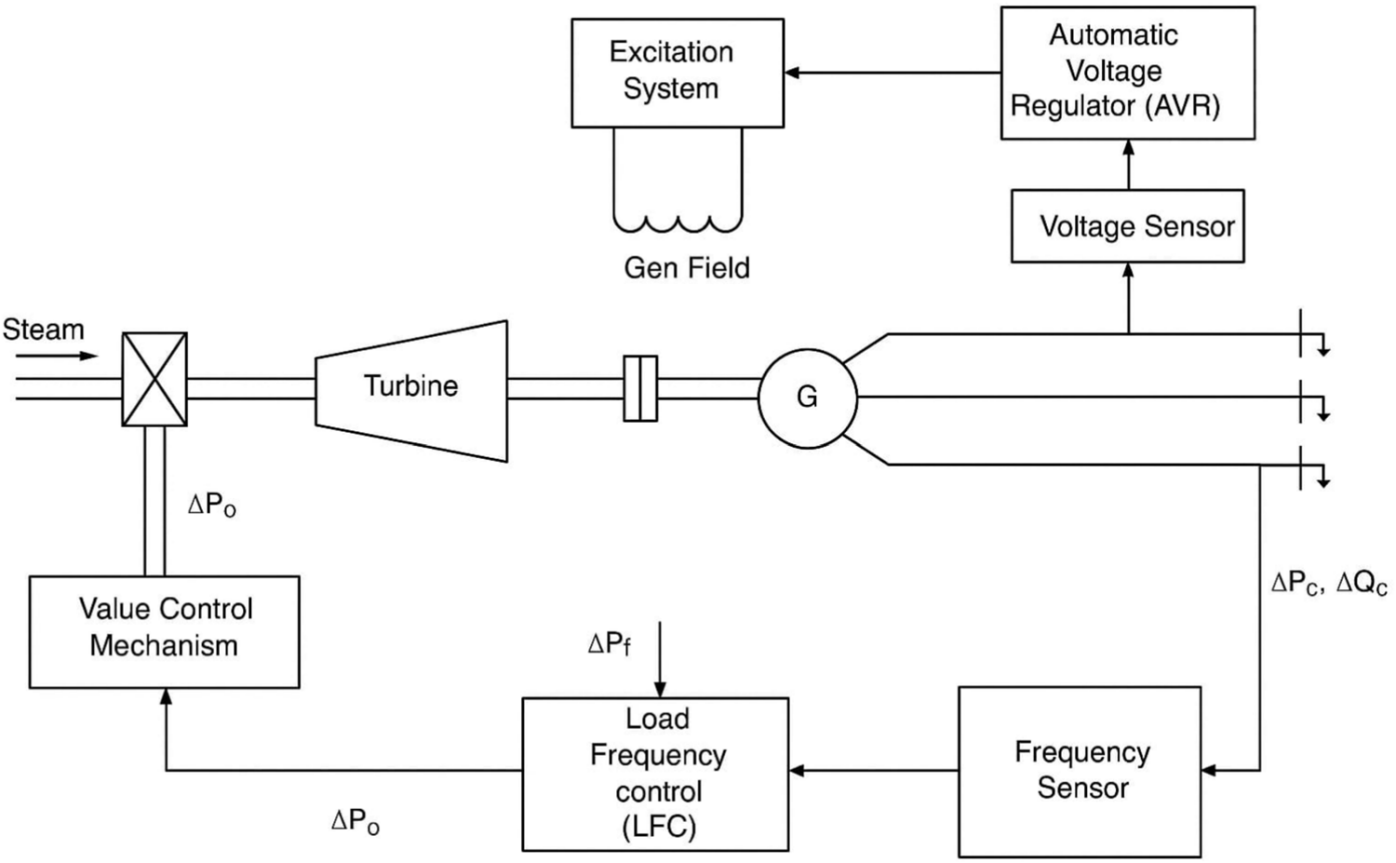
Schematic diagram of combined LFC and AVR system.

Power quality is a critical concern for both utilities and consumers, particularly due to the widespread use of sensitive electrical equipment. This has led to two primary challenges associated with non-linear loads: voltage fluctuation (VF) and load frequency control (LFC). Voltage fluctuation is predominantly influenced by reactive power flow, while load frequency is primarily affected by real power flow. As load demand varies randomly, it impacts both system frequency and tie-line power. Reactive power is closely tied to variations in voltage magnitude, whereas real power is more sensitive to changes in frequency. In an interconnected power system, each generator is typically equipped with a Load Frequency Control (LFC) system and an Automatic Voltage Regulator (AVR). The main objective of the LFC is to minimize transient deviations in frequency and tie-line power, ensure zero steady-state error, and enhance overall system stability. On the other hand, the AVR aims to maintain the terminal voltage of the synchronous generator at a desired level by adjusting its excitation voltage. This regulation of reactive power contributes significantly to the improvement of the power system’s steady-state stability. Figure 1 illustrates the schematic diagrams of both the LFC and AVR control loops. To maintain frequency and voltage within acceptable limits, these controllers are designed to respond to specific operating conditions and manage small fluctuations in load demand. Minor variations in rotor angle (δ), and thus in frequency, primarily affect real power, while reactive power is mainly governed by changes in voltage magnitude or generator excitation. Notably, the transient response of the excitation system is significantly faster than that of the prime mover, and the reduction in its time constant has negligible impact on the latter due to their distinct dynamic characteristics^1^.

To achieve greater accuracy in both dynamic and steady-state responses, the mutual interactions between the Load Frequency Control (LFC) and Automatic Voltage Regulator (AVR) loops are investigated using a combined Simulink model. This integrated model enables the study of coupling effects by incorporating both the mechanical and electrical dynamics under the control of the proposed controller. While many studies traditionally assume that the LFC and AVR loops operate independently, this assumption does not fully reflect the actual behavior of power systems. In reality, these loops are not entirely decoupled, and dynamic events can reveal noticeable interactions between them. In particular, the AVR and LFC loops are dynamically coupled through the generator’s internal electromotive force (EMF). Any change in excitation, controlled by the AVR, alters the terminal voltage, which in turn influences the real power output and, consequently, the system frequency—an aspect regulated by the LFC. However, the LFC operates on a much slower timescale compared to the fast response of the AVR. As a result, while the AVR has a noticeable effect on the LFC, the influence of LFC on the AVR is relatively minor. Therefore, these interactions must be taken into account when designing and analyzing control strategies to ensure accurate and robust system performance.

## Research gap

Existing approaches to Load Frequency Control (LFC) and Automatic Voltage Regulation (AVR) often rely on conventional PID controllers or standard optimization techniques that lack robustness, especially under system disturbances and parameter variations. Moreover, most studies focus on either LFC or AVR in isolation, without addressing their simultaneous optimization in single- and two-area interconnected systems. There is a clear need for a more effective controller design method that ensures stability and performance under a wider range of operating conditions.

The combined LFC-AVR control model plays a vital role by significantly improving system performance. Although it leads to an increase in frequency deviation (∆f) undershoot, it effectively reduces mechanical power deviation (∆Pm). The negative electrical power deviation (∆Pe) reveals that the AVR loop compensates for the reduced mechanical power, while voltage variations confirm AVR’s essential function in voltage regulation. These outcomes demonstrate that the integrated control approach is crucial for enhancing overall system stability and dynamic response, making it a key advancement over traditional isolated control methods.^2^

### Contribution

This study introduces a novel PID tuning approach using the Exponential Distribution Optimization (EDO) algorithm to simultaneously regulate frequency and voltage in both single- and two-area power systems. By leveraging the exploration–exploitation strengths of EDO and validating performance through ITSE and ITAE objective functions, the proposed method achieves faster convergence, improved dynamic response, and higher robustness under disturbances and ±50% time constant variations. Comparative results with Firefly Algorithm and Nonlinear Threshold-Accepting methods confirm the superiority of the proposed controller across all tested scenarios.

### Literature review

Several studies have focused on improving the dynamic response of load frequency control (LFC) and automatic voltage regulation (AVR) systems in power systems. In 2013^3^ improved the dynamic response of a combined LFC and AVR system using the Bacterial Foraging Optimization (BFO) algorithm to design PID controller parameters for a single-area power system, achieving better performance in terms of settling time and overshoot compared to the Ziegler method. In 2014^4^, applied the Coefficient Diagram Method to enhance robust LFC in a single-area power system with a wind farm, improving stability. In 2016^2^, used a neural network (NN) combined with fast traversal filters (FTF) to reduce steady-state error and speed up the dynamic response. In 2017^5^, introduced a high-performance HANFISC-TCSC that stabilizes an interconnected multi-area power system quickly. Another study in 2017^6^ used PID, fuzzy logic, and ANN-NARMA-L2 compensating schemes to mitigate errors from frequency and tie-line power disturbances in a two-area system, optimizing economic power generation. In 2018^7^, applied a Cascade PD-FOPID controller, optimized by the GHS algorithm, to a two-area thermal power plant with generation rate constraints, showing better performance than traditional PID and FOPID controllers. In 2019^8^, used the MFO algorithm to enhance the dynamic response of PID- and FOPID-controlled power systems. In the same year. In 2019^9^, demonstrated that the Firefly Algorithm (FA) outperforms Genetic Algorithm (GA) and Particle Swarm Optimization (PSO) in tuning PID parameters, improving frequency and voltage stability in a single-area power system. In 2020^10^, applied a Model Predictive Controller (MPC) for synchronized LFC and AVR in a three-area interconnected power system, improving the dynamic response and minimizing steady-state error. In 2021^11^, employed various meta-heuristic algorithms to design an LFC that enhances dynamic performance and ensures robustness against parametric uncertainties. In 2022^12^ introduced a Cascade Fuzzy PD-PID controller (CFPD-PIDN) for combined voltage and frequency control in electric vehicles and wind systems, showing improvements under various conditions. Another study in 2022^13^, investigated transient and steady-state performance in a multi-area interconnected power system (IPS), applying MPSO, LPBO, and AOA optimization techniques to improve PI-PD control strategies. In 2022^14^, a PI-FO + PD controller with fractional properties is presented to mitigate load frequency issues caused by changing demands and high renewable energy penetration, optimized using the dragonfly search algorithm (DSA) to enhance tracking efficiency and system stability under disturbances. In 2023^15^, optimized the FOPI-PIDD2 controller for voltage and frequency stabilization using the Dandelion Optimizer (DO) algorithm. In 2023^16^, applied the GBO-tuned FPIDD2 as a secondary regulator for LFC and AVR loops, achieving superior stability and robustness compared to traditional controllers under various operating conditions. In 2023^17^, a cascaded design of a model predictive controller with fractional order PID (MPC-FOPIDN) is presented to mitigate frequency oscillations caused by load disruptions, combining MPC’s predictive capabilities with fractional order control, optimized using the grasshopper optimization algorithm (GOA) to enhance load frequency control (LFC) and maintain system stability amidst fluctuations and uncertainties. In 2024^18^, a proactive frequency control (PFC) method is introduced for stabilizing electric power systems in distributed networks, utilizing a novel 4-DOF hybrid tilt model predictive controller (TMPC) with a 1 + PI-FOPI design, optimized by the tunicate swarm algorithm (TSA). In 2024^19^, a load frequency control method for maritime ships is presented, using a two-stage cascaded design with FOPI control and a 1 + TDN controller, optimized by the jellyfish search optimizer (JSO) to maintain frequency stability amidst renewable energy fluctuations and load variation. In 2024^20^, an adaptive tilt fractional order proportional integral derivative (TFOPID) controller optimized by the tunicate search algorithm (TSA) is introduced to enhance the stability and efficiency of multi-area power systems, addressing frequency fluctuations from renewable energy integration and load variation across regions with diverse power sources like photovoltaic (PV), wind, redox flow battery (RFB), hydro, and thermal plants. In 2024^21^, A novel, easily implementable method is introduced to enhance metacognitive algorithms by improving convergence speed, robustness, and avoidance of local minima; applied to the crow search optimization algorithm, this method—named diligent crow search optimization (DCSA)—significantly boosts performance in identifying solar cell parameters across various models and conditions, achieving a 98% increase in stability and sevenfold faster convergence. In 2025^22^, a study reviewed recent developments in load frequency control (LFC) for interconnected multi-area power systems, focusing on modern control strategies, optimization, and AI-based methods. In 2025^23^, A PIDN controller optimized by the artificial rabbit’s optimization algorithm enhances accuracy, stability, and robustness in electric furnace temperature control across varying conditions. In 2025^24^, A two-degree-of-freedom (2-DOF) PID controller optimized using quadratic interpolation optimization (QIO) significantly improves temperature control in continuous stirred tank reactors (CSTRs) by reducing steady-state error, settling time, and overshoot, outperforming various metaheuristic methods and recent approaches. In 2025^25^, A hybrid adaptive ant lion optimization (HAALO) algorithm combined with PI and FOPID controllers is proposed to reduce torque ripple and improve speed control in switched reluctance motors, achieving faster convergence and better accuracy than traditional methods. In 2025^26^, A novel multistage controller optimized by the diligent crow search algorithm (DCSA) improves pressure regulation in steam condensers, outperforming conventional methods by enhancing stability, response time, and reducing errors in nonlinear, disturbed systems.

## System modeling

Mutual effects of load frequency control LFC and Automatic Voltage Regulation (AVR) loops are investigated in a combined Simulink model of LFC and AVR loops to improve the accuracy in dynamic and steady-state responses. The recommended controller has been evaluated on a single-area model. Dynamic performance studies involving mechanical and electrical loops are carried out with the proposed PID controller to investigate the coupling effects of AVR and LFC loops^1^. The LFC is used to minimize the transient fluctuations in these variables and also to make sure that their steady-state error is zero and to improve the stability of the power system, while the main objective of AVR is to maintain the terminal voltage magnitude of a synchronous generator to a defined level by controlling its excitation voltage. It controls the reactive power and improves the steady-state stability of the power system^2^. LFC and AVR system modeling and parameter settings are illustrated in Tables 1 and 2.

**Table 1:** Literature review.

Year	Method/Algorithm	Application
2019	Firefly Algorithm (FA)	Single-area system
2020	Model Predictive Controller (MPC)	Multi-area system
2021	Meta-heuristic algorithms	LFC Area
2022	Cascade Fuzzy PD-PID	Electric vehicles & wind systems
2022	PI-FO + PD + DSA	LFC with renewable energy
2023	GBO-tuned FPIDD2	LFC & AVR
2024	TFOPID + TSA	Multi-area power systems
2025	PIDN + Artificial Rabbit’s Optimization	Electric furnaces
2025	HAALO + PI/FOPID	Switched reluctance motors

**Table 2:** LFC and AVR system modeling and parameter settings.

Model	Transfer function	Parameters	Description
Amplifier	$\frac{KA}{ 1+\tau AS}$	*KA* [10:400]*,* $\tau$ *A* [0.02:0.1s]	Gains and time constants of amplifier, exciter, generator, and sensor
Exciter	$\frac{KE}{ 1+\tau ES}$	*KE,* $\tau$ *E (Very small)*	
Generator	$\frac{KG}{ 1+\tau GS}$	*KG* [0.7:1]*,* $\tau$ *G* [1.0:2.0s]	
Sensor	$\frac{KR}{ 1+\tau RS}$	*KR,* $\tau$ *R* [0.01:0.06s]	
Governor	$\frac{Kg}{ 1+\tau gS}$	$\tau g,$ *Kg*	Gains and time constants of governor, turbine, and generator/load
Turbine	$\frac{KT}{ 1+\tau TS}$	*KT,* $\tau$ *T* [0.2: 2.0s]	
Inertia/Load	$\frac{Kl}{ 1+\tau lS}$	*Kl,*$\tau l$	
–	–	*B*	Frequency bias coefficients
–	–	*R*	Governor speed regulation

In general, investigations assume that the LFC and AVR loops do not interact. However, the AVR and LFC loops are not truly non-interacting. In practice, various interactions between these two control loops arise during dynamic problems. AVR loops with more rapid actions change the magnitude of the generator emf by modifying the generator’s excitation winding. Because internal EMF controls the level of real power, changes in AVR must be noticed in the LFC loop.

The product of the synchronizing power coefficient *Ps* and the change in the power angle *∆δ* results in a minor change in real power *δ*. If we consider the minor effect of voltage on real power *∆Pe.*

1 $$\Delta {\text{Pe }} = {\text{ Ps }}\Delta \delta \, + {\text{ {K2 E}}^{\prime}}$$

where K2 denotes the change in electrical power caused by a minor change in stator emf E’.

2 $$\Delta {\text{Vt }} = {\text{ K5}}\Delta \delta \, + {\text{ {K6 E}}^{\prime}}$$

where *K5* reflects the change in terminal voltage for a minor change in rotor angle at constant stator emf and *K6* reflects the change in terminal voltage for a minor variation in stator emf at constant rotor angle.

3 $$E{\prime} =\frac{KG}{ 1+\tau G}(Vf - K4 \Delta \delta )$$

the stator emf by incorporating the influence of rotor angle into the generator field transfer function.

## Controller

For more than 50 years, industry firms have successfully used the PID controller, which is a simple and dependable controller that can provide outstanding control performance regardless of the dynamic characteristics of the process plant^27^. The proportional integral derivative (PID) controller is among the most widely used commercially available controllers. The proportional integral derivative (PID) controller is the most popular among the available alternatives. The PID controller is distinguished from other types of controllers by its reliable performance across a wide range of operating situations and its straightforward structural design^28^. The PID controller operates with three control parameters: proportional, integral, and derivative gains. Several heuristic optimization strategies have been developed to improve the response of the combined LFC and AVR system. These approaches have garnered respect among experts all across the globe^29^. The PID controller improves dynamic response by minimizing or eliminating steady-state faults. The derivative controller enhances transient response by adding a finite zero to the open-loop plant transfer function. The integral controller adds a pole at the origin, increases the system type by one, and decreases the steady-state error caused by a step function to zero^30^. The transfer function of the PID controller is:

4 $$Gc\left(s\right)={K}_{P}+\frac{{K}_{I}}{ S}+{K}_{D} s$$

## Optimization techniques

Optimization approaches use artificial intelligence or evolutionary computation to fine-tune the controller parameters. EDO is used as an optimization tool in conjunction with the combined LFC and AVR systems.

### Exponential distribution optimizer

**Fig. 2:**
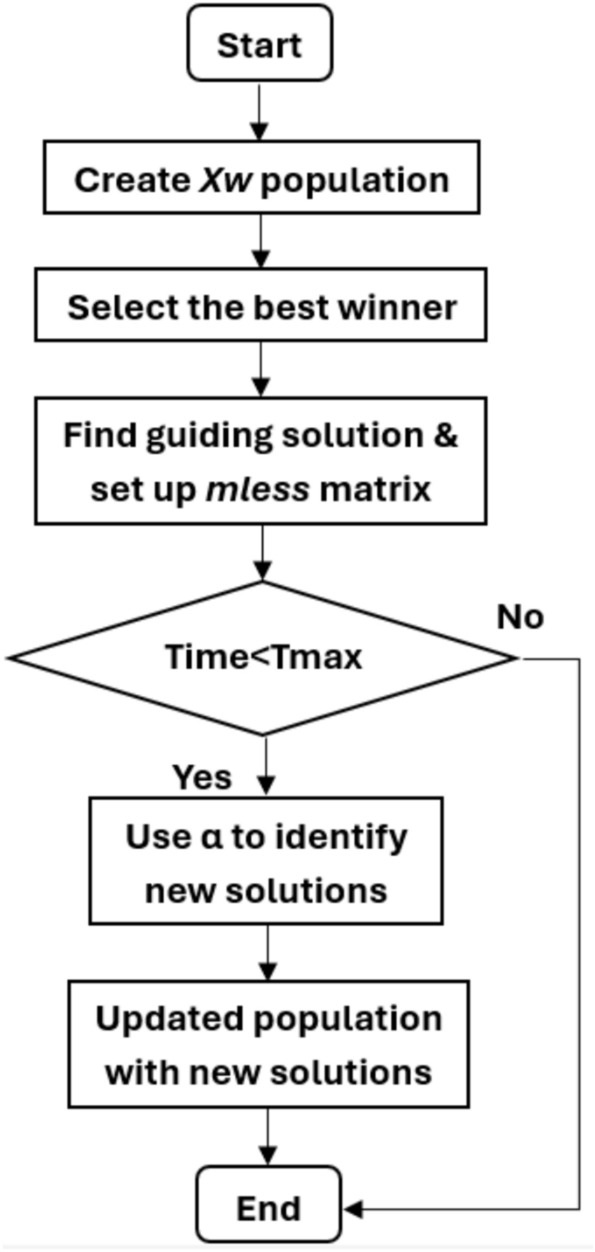
The flowchart of the EDO optimizer.

In 2023, the Exponential Distribution Optimization Algorithm (EDO) was published. It has a high explorative capability by using two randomly chosen winners to create two individuals with similar characteristics to uncover potential areas of the search space. It is simple to implement and has both explorative and exploitative capabilities. Statistical analysis indicates that the proposed EDO is superior with 95% confidence^31^. Industrial applications frequently use the proportional integral derivative (PID) controller because of its simplified structure and ease of implementation. While the PID controller performs effectively in the majority of systems, customized versions of the PID control structure have demonstrated better performance in numerous control systems, including the AVR-LFC interconnected power system. EDO is used to achieve better results as an effective tool for solving optimization algorithm problems and the most advanced optimization algorithm method, demonstrating its efficiency in stabilizing steady state error values under different disturbances in the combined LFC and AVR system, so it is used to find the optimum PID controller parameters, while the objective function enhances stability and response. The outcomes are compared to several optimization techniques to determine the optimum performance and stability of the combined LFC and AVR system. Exponential Distribution Optimization Algorithm (EDO), which is characterized by its simplicity of implementation and its explorative and exploitative capabilities, is known for its high exploration capability. It achieves this by using two randomly selected winners to develop two other individuals who share various characteristics with these winners in order to uncover additional areas of the search space. Research on statistics indicates that, with a 95% confidence level, the suggested EDO is preferable^31^. The exponential distribution is a continuous distribution that deals with the amount of time it takes for an event to occur, the flowchart of the EDO optimizer is shown in Fig. 2.

The computational feasibility of the proposed EDO-based optimization was carefully examined. Owing to its simple operators and limited control parameters, EDO imposes a relatively low computational burden compared with other metaheuristic algorithms such as GA or PSO. All simulations were executed on a standard desktop computer (Intel Core i5, 1.8 GHz, 8 GB RAM), with convergence achieved well within practical computation times. This demonstrates that the method can be implemented without requiring high-performance computing resources. In terms of scalability, the algorithm was successfully applied to both single- and two-area interconnected models, showing stable convergence and robustness. Nevertheless, as with most population-based techniques, EDO may face challenges such as premature convergence in very large-scale multi-area systems or highly complex nonlinear models, which constitutes a potential limitation and an avenue for future investigation.

#### The memoryless property

It can be described by Eqs. (5)–(7).

5 $${\text{P}}\left( {{\text{y }} > \left. {{\text{s}} + {\text{t}}} \right| {\text{y }} \ge {\text{s}}} \right) = {\text{P}}\left( {{\text{y}} > {\text{t}}} \right) \, ,{\text{ if t }} > \, 0{\text{ and s }} > \, 0$$

y: A random variable indicates waiting time until an event occurs.

6 $$\mu = \frac{1}{2}\left( {mless^{time} \,i\, + \,Xg^{time} } \right)/2,\,Xg^{time} :\,{\text{the}}\,{\text{guiding}}\,{\text{solution}}$$

*mless: a matrix which stores the current new solutions regardless of their current fitness.*

7 $${\text{Xg}}^{{{\text{time}}}} = \, \left( {{\text{Xw}}^{{{\text{time1}}}} + {\text{Xw}}^{{{\text{time2}}}} + {\text{Xw}}^{{{\text{time3}}}} } \right){\text{best }}/{3}$$

Xw: a population consists of N randomly generated solutions with widely varying values.

#### The EDO exploitation phase

It relays on a memoryless property, standard variance, and means to update the new solution, and described by Eqs. (8).

8 $${V}^{time+1}i=\left\{\begin{array}{c}p.{(mless}^{time}i -{\sigma }^{2})+q.X{g}^{time} if X{w}^{time}i={mless}^{time}i \\ q.({(mless}^{time}i-{\sigma }^{2})+log(\theta ) X{w}^{time}i , otherwise\end{array}\right.$$

$$p={(r)}^{10} , q={(r)}^{5}$$

*p* and *q* are variables that can be changed.

$\theta$ is a random number in the range [0, 1].

*r* is an integer created randomly in the range [-1, 1].

#### The EDO exploration phase

The exploration is created using two random winners from the initial population (*Xw rand*1, *Xw rand*2) and updated using:

$${V}^{time+1}i=X{w}^{time}i - {E}^{time} + (c.Y1 + (1-c).Y2)$$

9 $${E}^{time}=\frac{1}{N}. \sum_{i=1}^{N}X{w}^{time}j,i , j = 1, 2, ....., m$$

*N*: The population size.

*m*: The dimension of the problem.

*Tmax*: The total number of iterations.

*E*^*time*^*:* The average of all solutions acquired in the initial population.

*e:* is an adjusted parameter representing the information exchange ratio between the *Y1* and *Y2* vectors.

The Exploration can be described by Eqs. (10)–(14).

10 $$Y1 \, = \, E \, - \, W1 \, + \, W2$$

11 $$Y2 \, = \, E \, - \, W2 \, + \, W1$$

12 $$W1 \, = \, E \, - Xw \, rand1$$

13 $$W2 \, = \, E \, {-}Xw \, rand2$$

14 $$m=\frac{1-time}{ Tmax} , e=m x r$$

#### EDO optimizer

**Figure:**
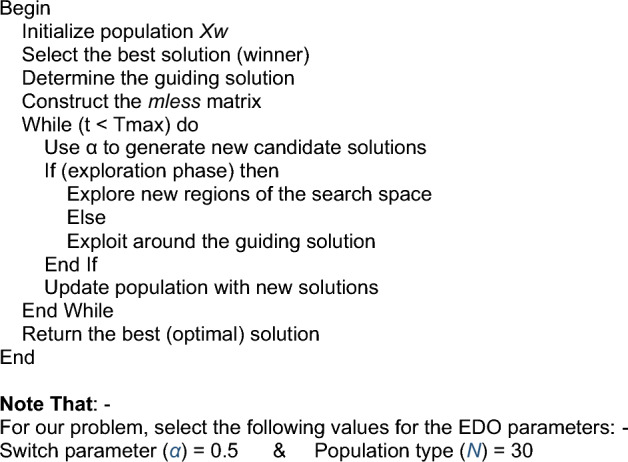
Pseudo-code of EDO Algorithm.

## Objective function

The term “objective function” aims to better stability and faster time response. The optimization techniques begin with the selection of random integers inside the constraints to modify controller parameters until optimal solutions or maximum iterations are obtained, which influence the response and stability of a combined LFC and AVR system. The proposed objective functions as follows^32^:

- Integrated Time-weighted-Squared-Error (ITSE)^33^:
  15 $$F\left(t\right)={\int }_{0}^{t}{\left(e\right)}^{2}dt$$
- Integral Time-Weighted-Absolute Error (ITAE)^33^:
  16 $$F\left(t\right)={\int }_{0}^{t} t \left| e \right| dt$$

## Case I – single area IPS model

**Fig. 3:**
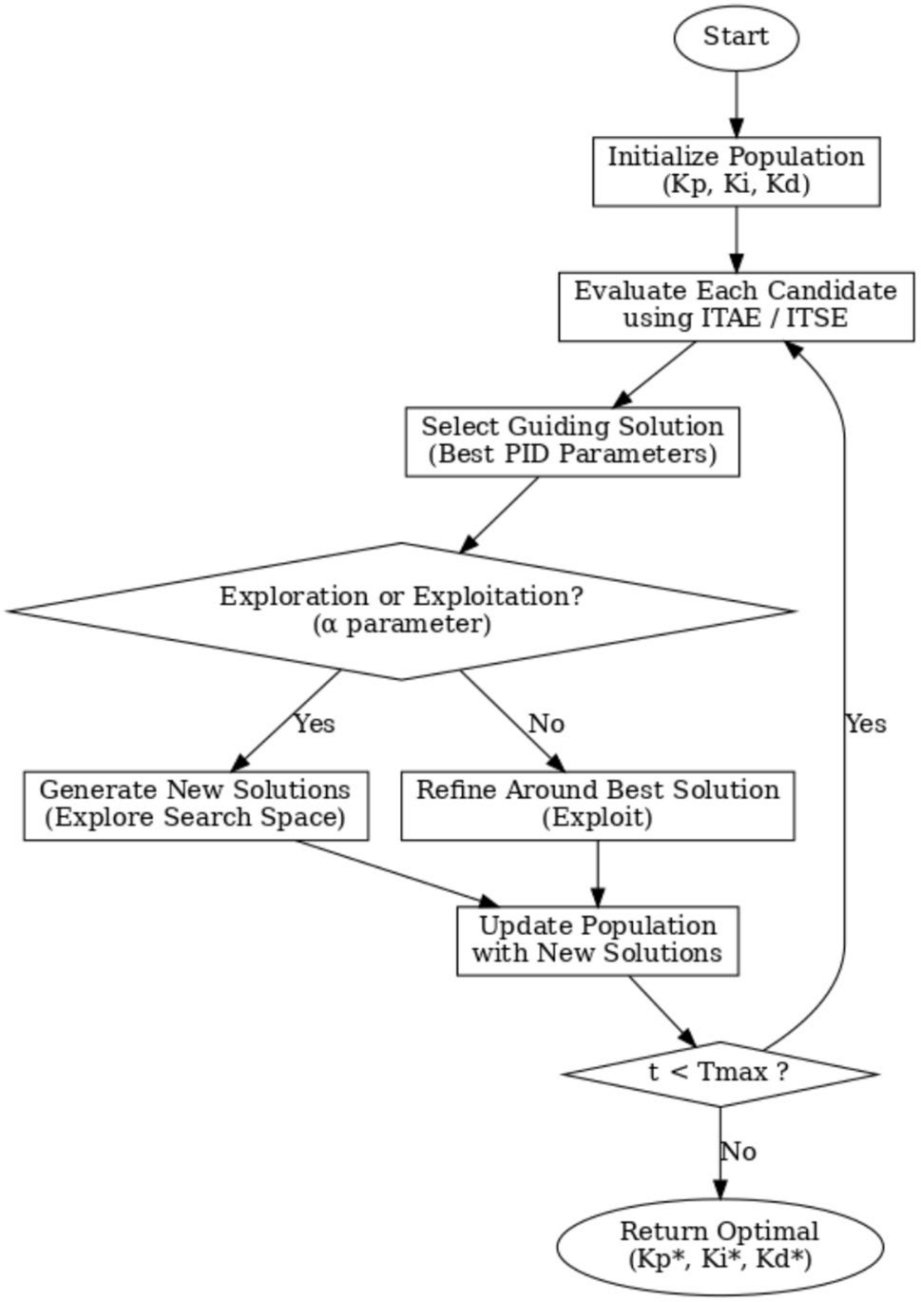
A visual block diagram showing the EDO optimizer integration with the PID controller.

The implementation of the combined LFC and AVR system for a single area model is shown in Fig. 3, which uses a PID controller to improve the system’s dynamic performance.

## Simulation results

**Fig. 4:**
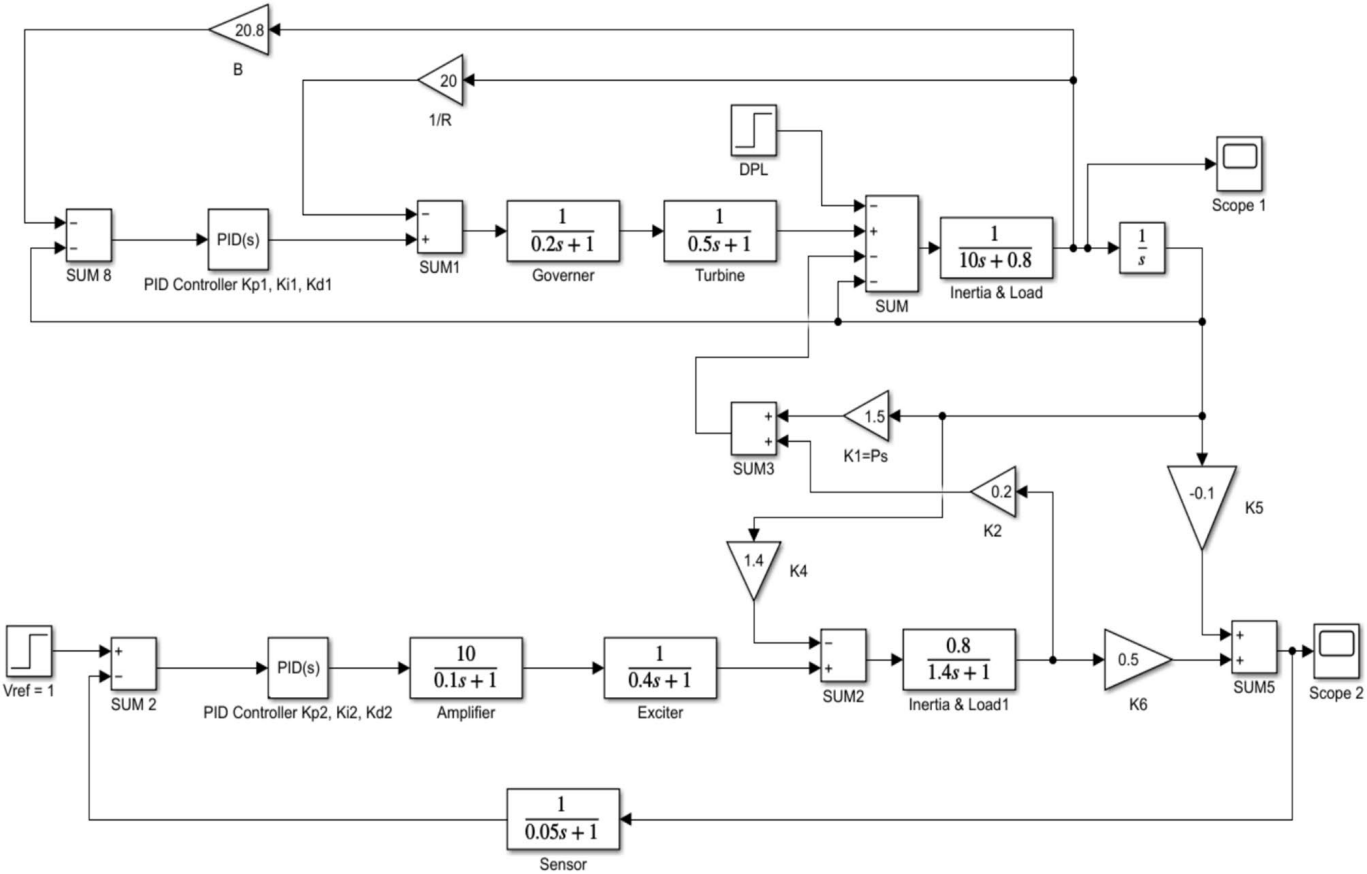
MATLAB/simulation model of LFC with AVR system with PID controller.

Using MATLAB *R2021a, 64-bit* /SIMULINK runs on a machine with an Intel Core i5 8^th^ Gen. processor running at 1.8 GHz and 8 GB of RAM. Figure 4 shows the implementation of the previously mentioned combined LFC and AVR system.

### Transient response

Applying the EDO optimization technique to tune the PID controller parameters, as shown in Table 3, which is used to enhance the dynamic performance of the combined LFC and AVR system, as well as comparing the frequency and voltage deviation characteristics for the proposed EDO-PID controller and FA-PID controller ^9^ using two types of objective functions, ITSE and ITAE, as mentioned in Table 4.

**Table 3:** Optimization techniques response using PID controller.

Optimization		Kp1	Ki1	Kd1	Kp2	Ki2	Kd2
EDO	ITSE	2.9817	2.8829	2.9775	2.6885	0.5598	0.7357
	ITAE	2.9924	2.8389	2.8204	2.6693	0.5567	0.7410
FA^9^	ITAE	2.9931	2.9972	2.4862	2.6872	0.5717	0.7283
	ITSE	2.9850	2.9916	2.4238	2.8715	0.5821	0.7764

**Table 4:** Frequency/voltage deviation characteristics and PID tuning using EDO and FA.

	Freq				Voltage			
	Md (p.u)	tp (sec.)	ts (sec.) ± 5%	Ess (p.u)	Mp (p.u)	tp (sec.)	ts (sec.) ± 2%	Ess (p.u)
EDO-ITSE	− 5.949 × 10^–3^	0.6842	8.70	− 2.725 × 10^–4^	1.0069	0.7672	1.418	1.1 × 10^–4^
FA-ITSE^9^	− 6.932 × 10^–3^	0.6719	8.23	− 2.803 × 10^–4^	1.0162	0.7526	1.385	− 1.3 × 10^–4^
EDO-ITAE	− 6.115 × 10^–3^	0.6847	8.84	− 2.824 × 10^–4^	0.9998	0.7675	1.415	3 × 10^–6^
FA-ITAE^9^	− 6.674 × 10^–3^	0.6874	8.73	− 2.798 × 10^–4^	1.0129	0.7705	1.422	− 1 × 10^–4^

**Fig. 5:**
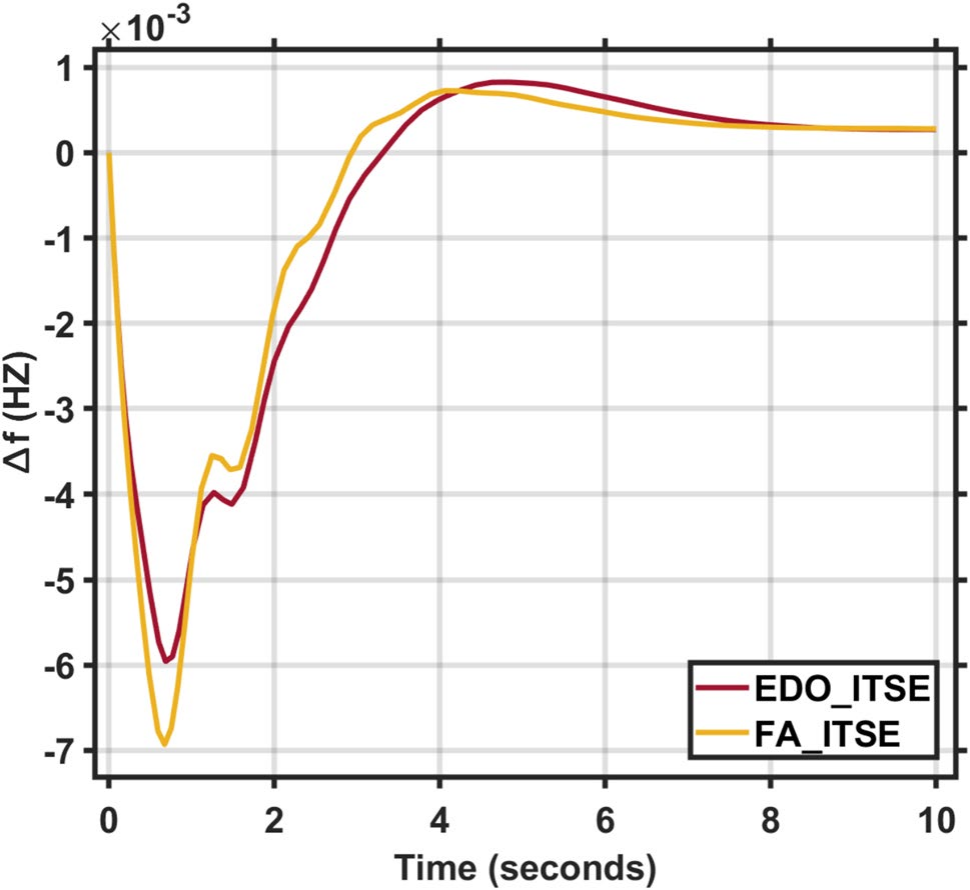
LFC response with PID FA and EDO based using ITSE.

**Fig. 6:**
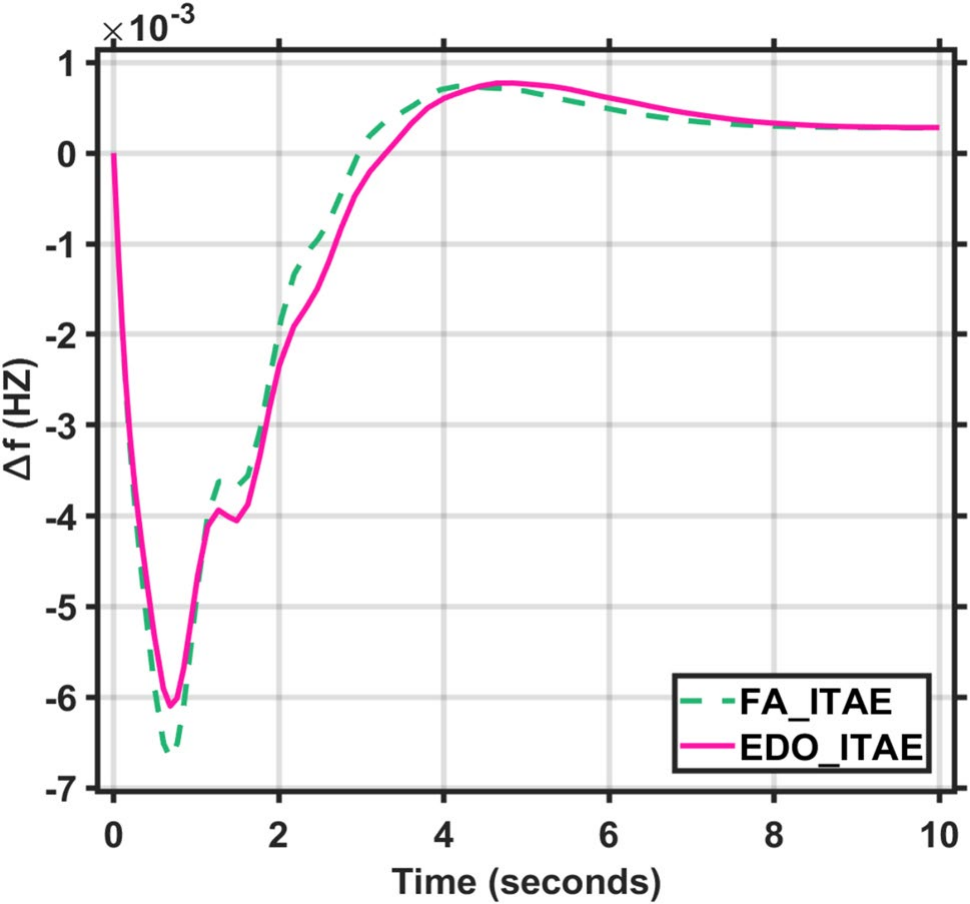
LFC response with PID FA and EDO based using ITAE.

**Fig. 7:**
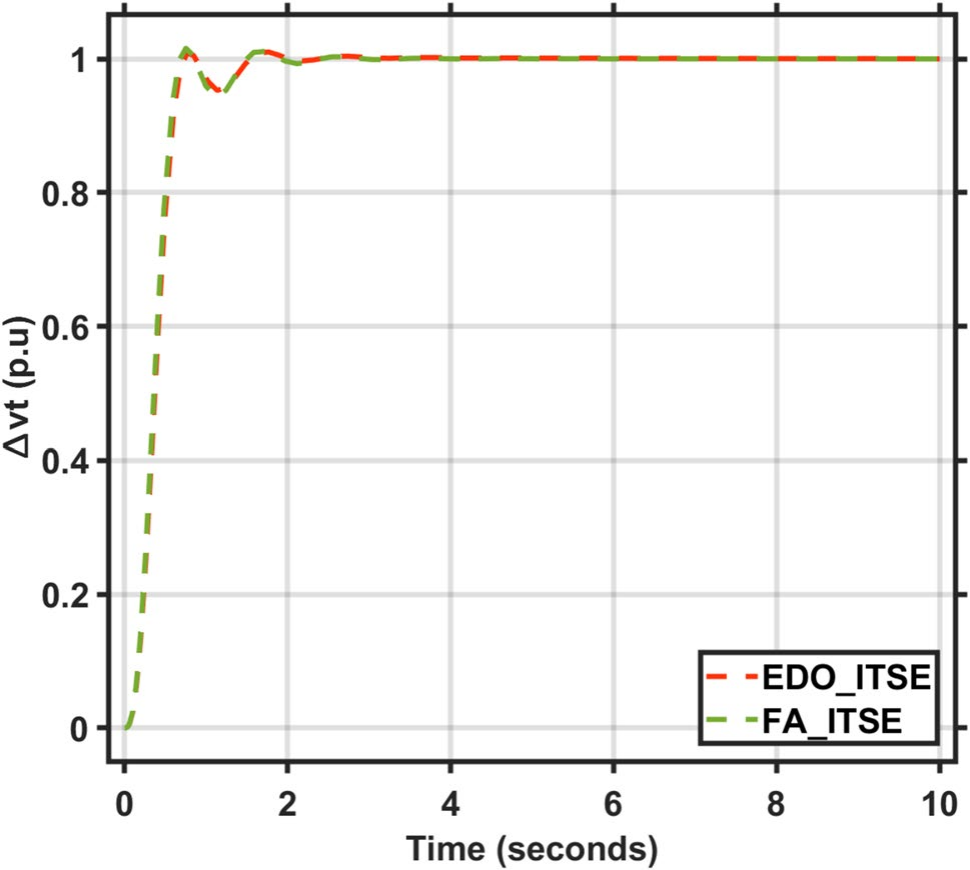
AVR response with PID FA and EDO based using ITSE.

**Fig. 8:**
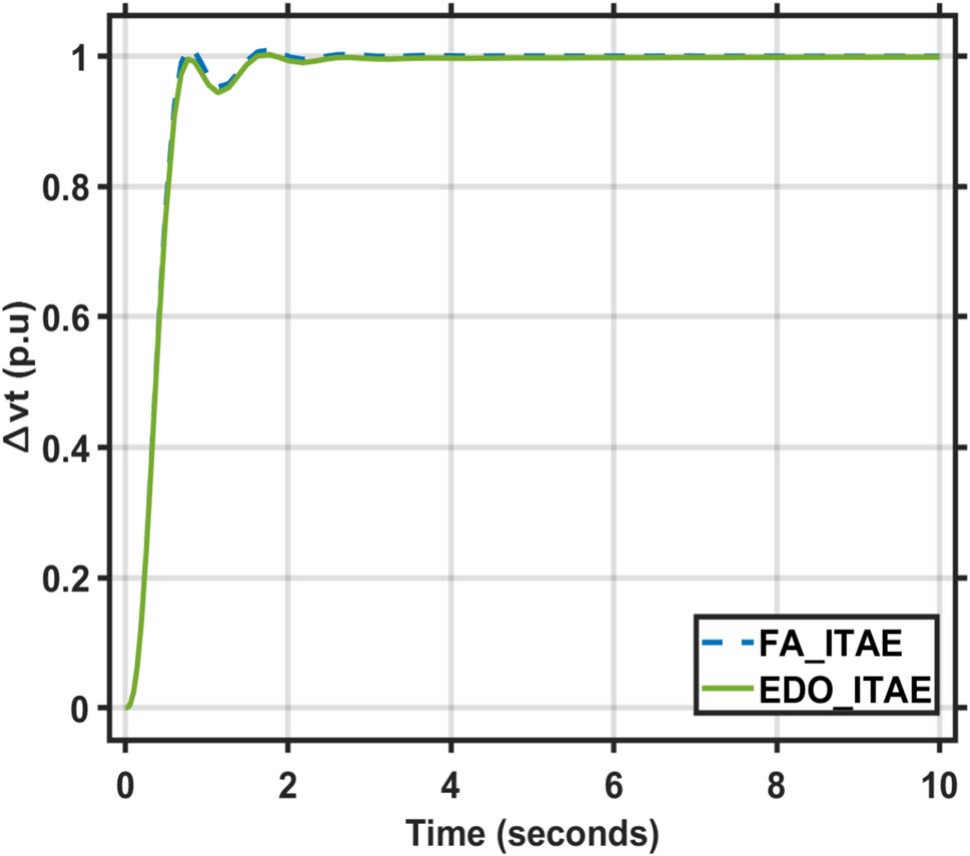
AVR response with PID FA and EDO based using ITAE.

The EDO-PID controller scheme improved LFC response undershoots by 16.52% compared to the FA controller, steady-state error by 2.86% better than the FA controller, as shown in Figs. 5 and 6, and AVR response improved by 1.31% overshoots compared to the FA controller, steady-state error by 33.3% better than the FA controller, as shown in Figs. 7 and 8.

### Robustness analysis

**Fig. 9:**
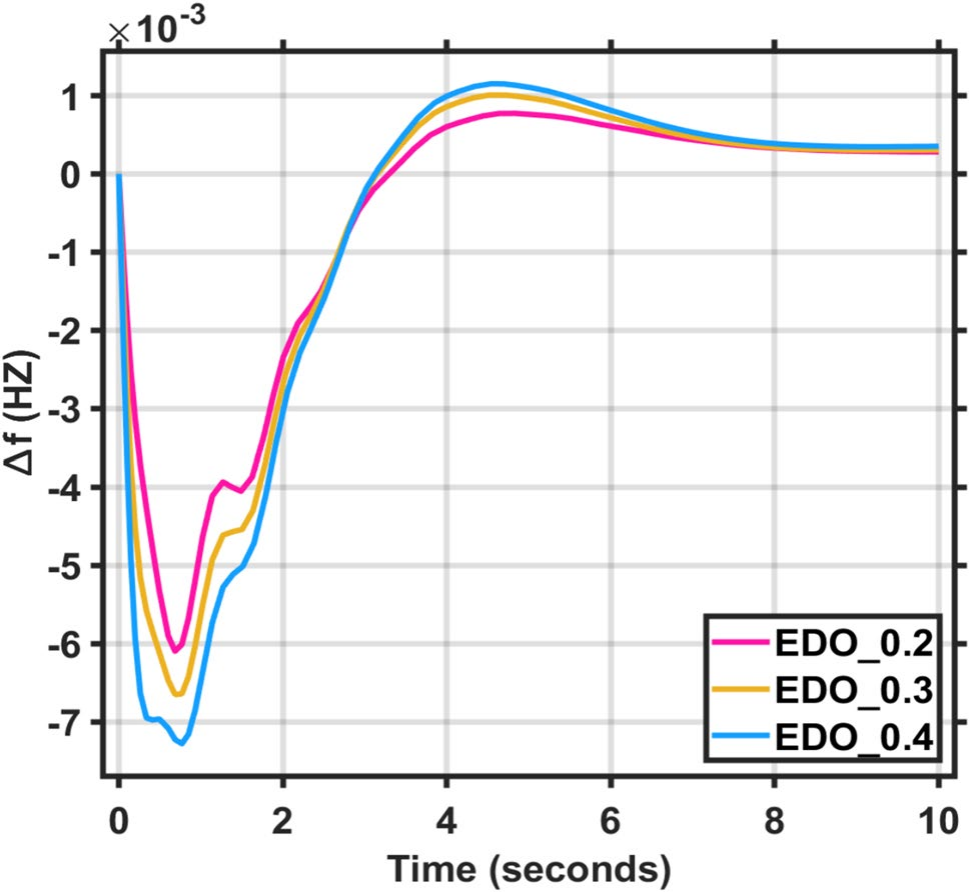
LFC response with PID EDO based under different step function (∆Pl).

**Fig. 10:**
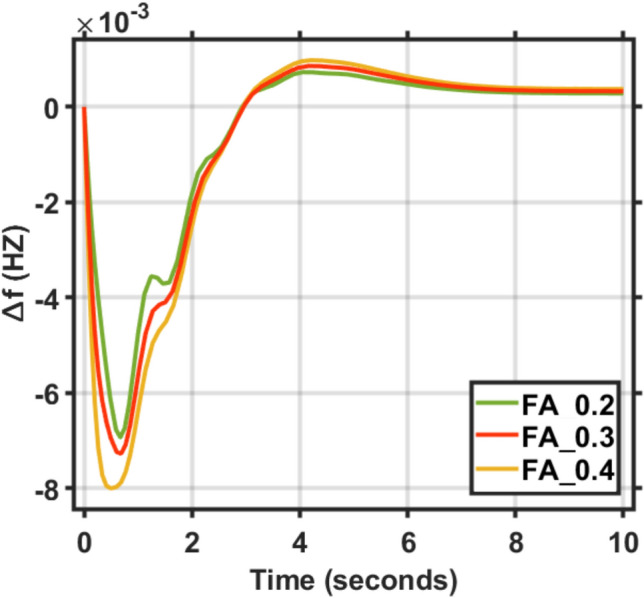
LFC response with PID FA based under different step function (∆Pl).

In this section, two types of tests applied on the combined LFC and AVR system for a single area to demonstrate its reliability and effectiveness. Firstly, the deviation in load, represented by different values of the step function (∆Pl), ranging from zero to 0.2, 0.3, and 0.4, was applied to evaluate the dynamic response of the combined LFC and AVR system under the tuned proposed EDO-PID controller in comparison to the FA controller. The frequency and voltage deviation characteristics, along with PID tuning using EDO and FA, are detailed in Table 5 and the response of the system under deviation in load is shown in Figs. 9 and 10. The results show that the AVR response with the EDO-PID controller exhibited 1.31%, 1.3%, and 1.31% better overshoots than the FA controller, along with a faster dynamic response and improved steady-state error, which reached 33.3%, 2.61%, and 4.26%, respectively.

**Table 5:** Frequency/Voltage deviation characteristics and PID gains under different step function (∆Pl).

ITAE	Freq				Voltage			
	Md (p.u)	tp (sec.)	ts (sec.) ± 5%	Ess (p.u)	Mp (p.u)	tp (sec.)	ts (sec.) ± 2%	Ess (p.u)
EDO (∆Pl = 0.2)	− 6.115 × 10^–3^	0.6847	8.84	− 2.824 × 10^–4^	0.9998	0.7675	1.415	3 × 10^–6^
EDO (∆Pl = 0.3)	− 6.684 × 10^–3^	0.6847	8.85	− 3.309 × 10^–4^	0.9999	0.7674	1.415	− 7.27 × 10^–5^
EDO (∆Pl = 0.4)	− 7.265 × 10^–3^	0.7674	8.72	− 3.785 × 10^–4^	0.9999	0.7674	1.415	− 4.23 × 10^–5^
FA (∆Pl = 0.2)^9^	− 6.674 × 10^–3^	0.6874	8.73	− 2.798 × 10^–4^	1.0129	0.7705	1.422	− 1 × 10^–4^
FA (∆Pl = 0.3)^9^	− 7.273 × 10^–3^	0.6873	8.20	− 3.255 × 10^–4^	1.01294	0.7704	1.415	− 1.9 × 10^–4^
FA (∆Pl = 0.4)^9^	− 8.002 × 10^–3^	0.4940	8.17	− 3.723 × 10^–4^	1.01299	0.7704	1.422	− 1.8 × 10^–4^

**Fig. 11:**
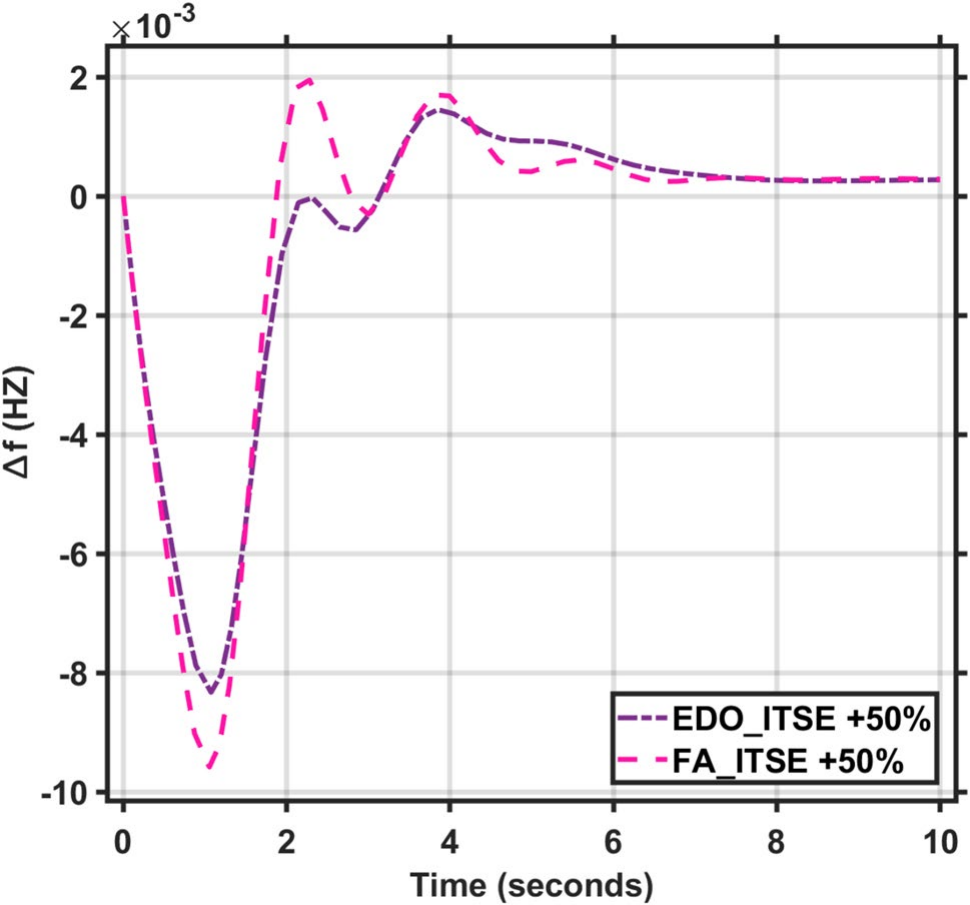
LFC response with PID FA and EDO based with variation + 50% time constants.

**Fig. 12:**
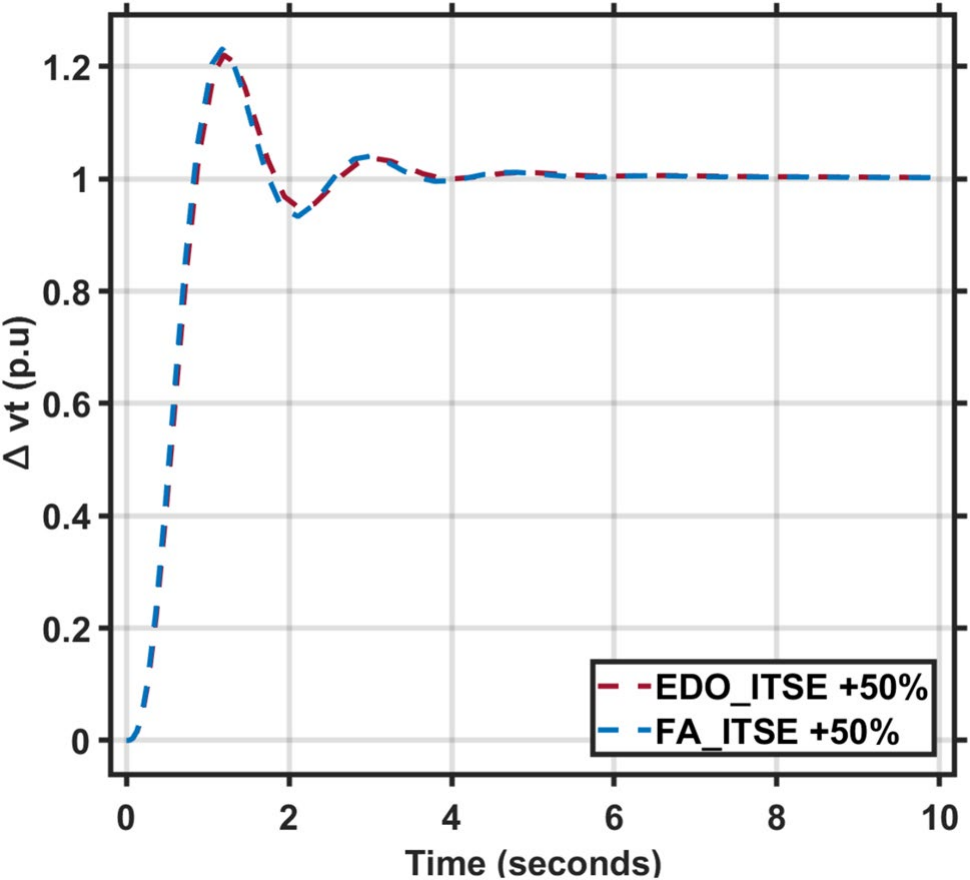
AVR response with PID FA and EDO based with variation + 50% time constants.

**Fig. 13:**
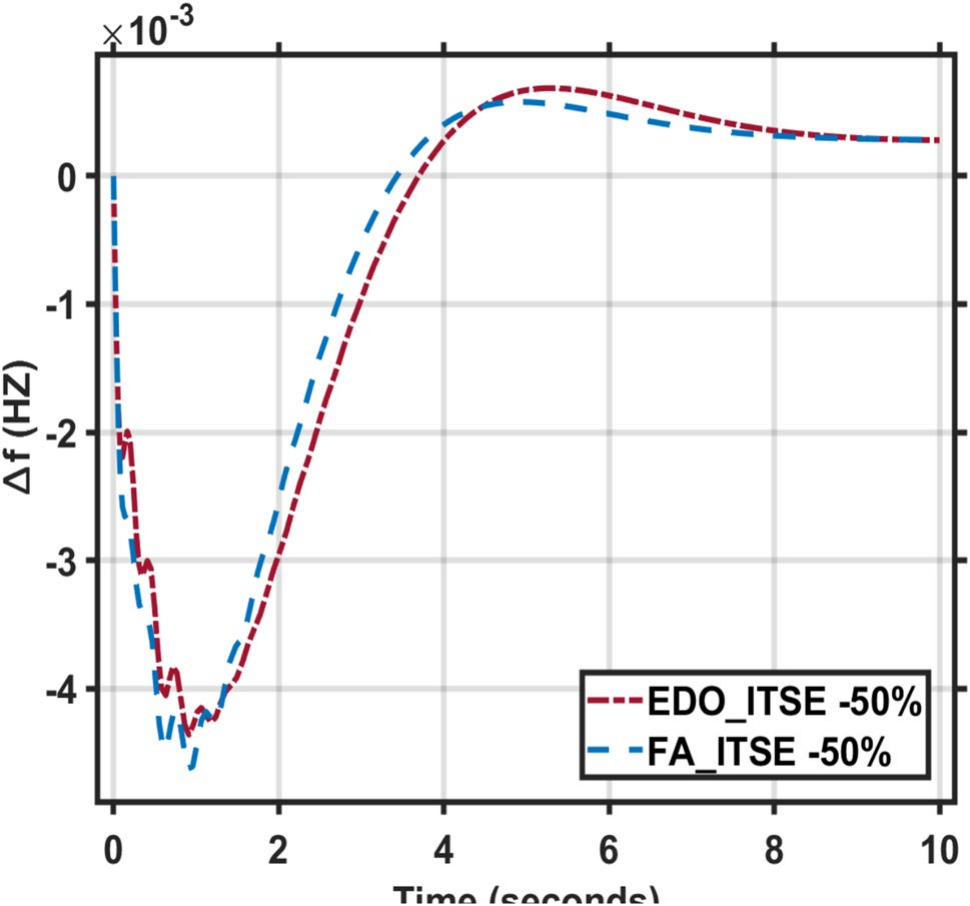
LFC response with PID FA and EDO based with variation − 50% time constants.

**Fig. 14:**
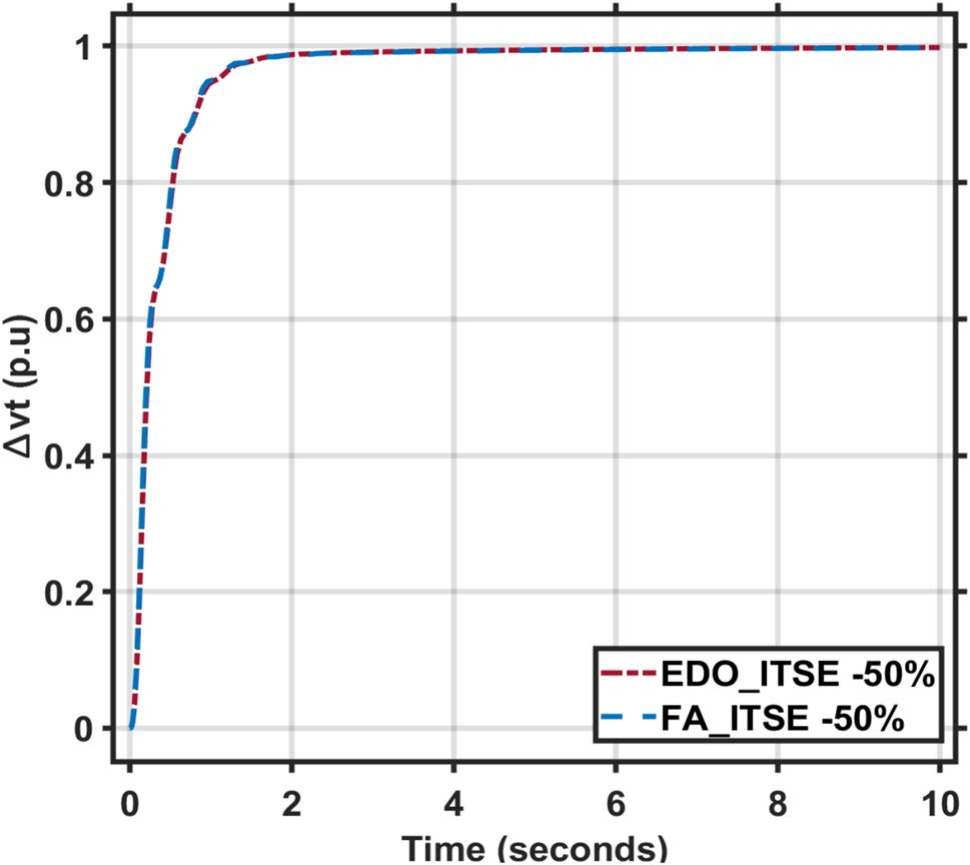
AVR response with PID FA and EDO based with variation − 50% time constants.

Secondly, the time constants for the combined AVR and LFC models fluctuate between -50% and + 50% of their nominal values. The dynamic response characteristics, including maximum overshoot, rise time, settling time, and steady-state error, for these time constant variations are presented in Tables 5 and 6. Figures 11, 12, 13 and 14 illustrate the system’s response under these changes. These Figures and Tables highlight the robustness of the combined AVR and LFC system and demonstrate the ability of the EDO-PID controller to enhance stability and response during load variations.

**Table 6:** The combined AVR and LFC system dynamic response with variation + 50% in system time constant parameters ($\tau$ *A,* $\tau$ *E,* $\tau$ *G,* $\tau$ *s,* $\tau$ *R,* $\tau$ *g, *$\tau l$*,* $\tau$ *T*).

ITAE	Freq				Voltage			
	Md (p.u)	tp (sec.)	ts (sec.) ± 5%	Ess (p.u)	Mp (p.u)	tp (sec.)	ts (sec.) ± 2%	Ess (p.u)
EDO	− 8.309 × 10^−3^	1.073	7.65	− 2.74 × 10^−4^	1.2168	1.199	3.42	− 2.197 × 10^−3^
FA	− 9.579 × 10^−3^	1.053	7.79	− 2.84 × 10^−4^	1.2313	1.177	3.33	− 2.23 × 10^−3^

Figure 7 indicates that the LFC response with EDO-PID controller which had 9.14%, 8.81%, and 10.14% better undershoots than the FA controller, as well as faster dynamic response and better steady-state error.

It is observed that the EDO control scheme demonstrated 15.28% better undershoots in the LFC response compared to the FA controller, with a 3.65% improvement in steady-state error. Additionally, the AVR response showed a 1.19% improvement in overshoots and a 1.83% reduction in steady-state error compared to the FA controller with variation + 50% in system time constant parameters (Table 7).

**Table 7:** The combined AVR and LFC system dynamic response with variation − 50% in system time constant parameters ($\tau$ A, $\tau$ E, $\tau$ G, $\tau$ s, $\tau$ R, $\tau$ g, $\tau l$, $\tau$ T).

ITSE	Freq				Voltage			
	Md (p.u)	tp (sec.)	ts (sec.) ± 5%	Ess (p.u)	Mp (p.u)	tp (sec.)	ts (sec.) ± 2%	Ess (p.u)
EDO	− 4.36 × 10^–3^	0.911	9.125	− 2.78 × 10^–4^	0.9975	10	1.608	2.5 × 10^–3^
FA	− 4.63 × 10^–3^	0.901	8.765	− 2.81 × 10^–4^	0.9977	10	1.538	2.3 × 10^–3^

It is observed that the EDO control scheme showed 6.17% better undershoots in the LFC response compared to the FA controller, with a 1.15% improvement in steady-state error. The AVR response also exhibited a 0.02% improvement in overshoots and better steady-state error compared to the FA controller. The robustness analysis indicates that the EDO-PID output is adequately resilient to changes in load with variation -50% in system time constant parameters.

### Convergence plots

**Fig. 15:**
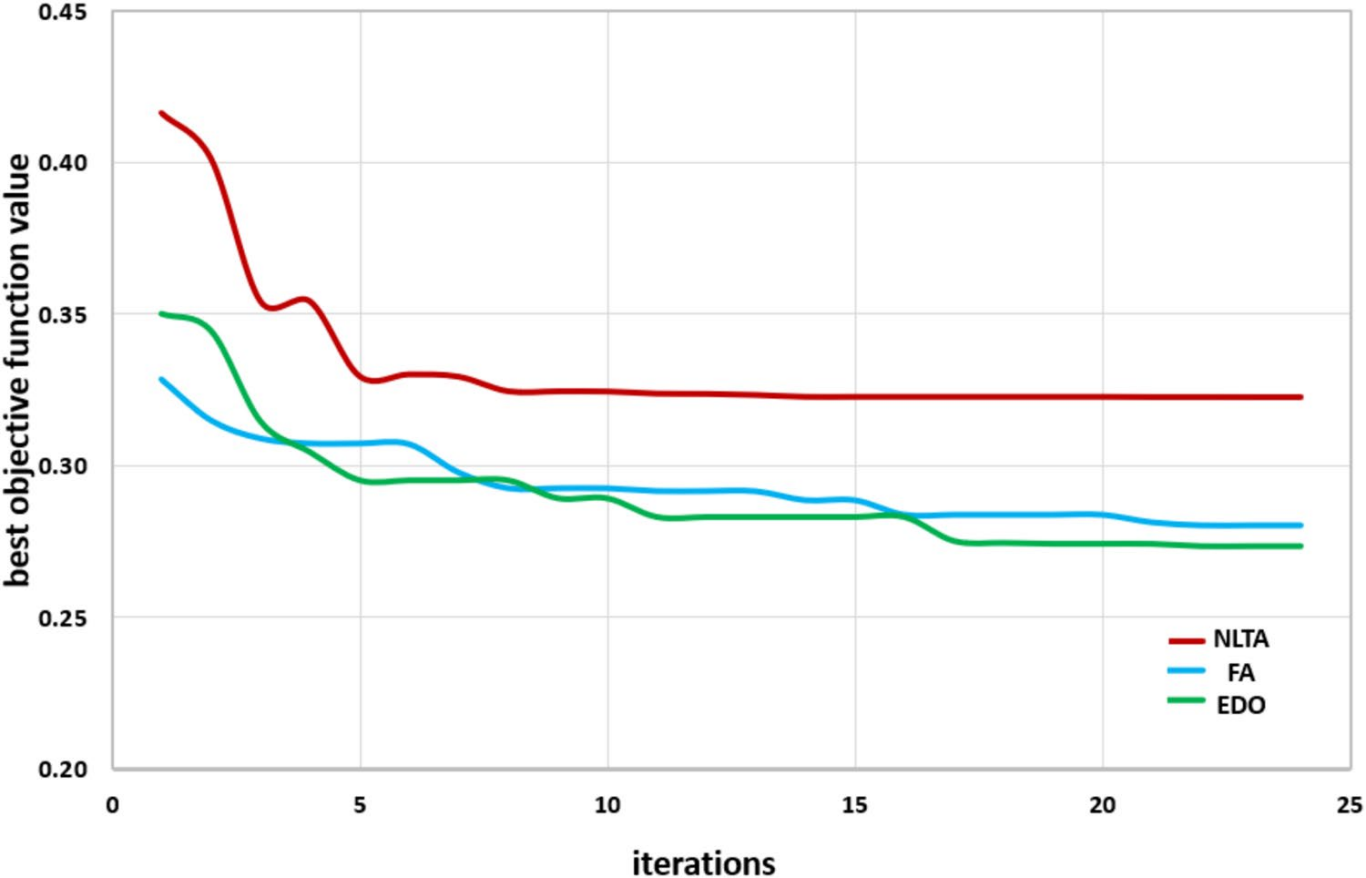
Best objective function value VS. iteration numbers with 10 runs of EDO, FA, NLTA.

To evaluate the convergence behavior of the EDO and assess the robustness of the algorithm, the EDO-based optimization method was executed ten times using randomly generated initial conditions. The outcomes, illustrated in Fig. 15, display the best performing particles across iterations. This analysis confirms that 100 iterations are sufficient and highlights the method’s capability to efficiently and accurately optimize PID parameters for the given power system model.

## Case II two area IPS model

**Fig. 16:**
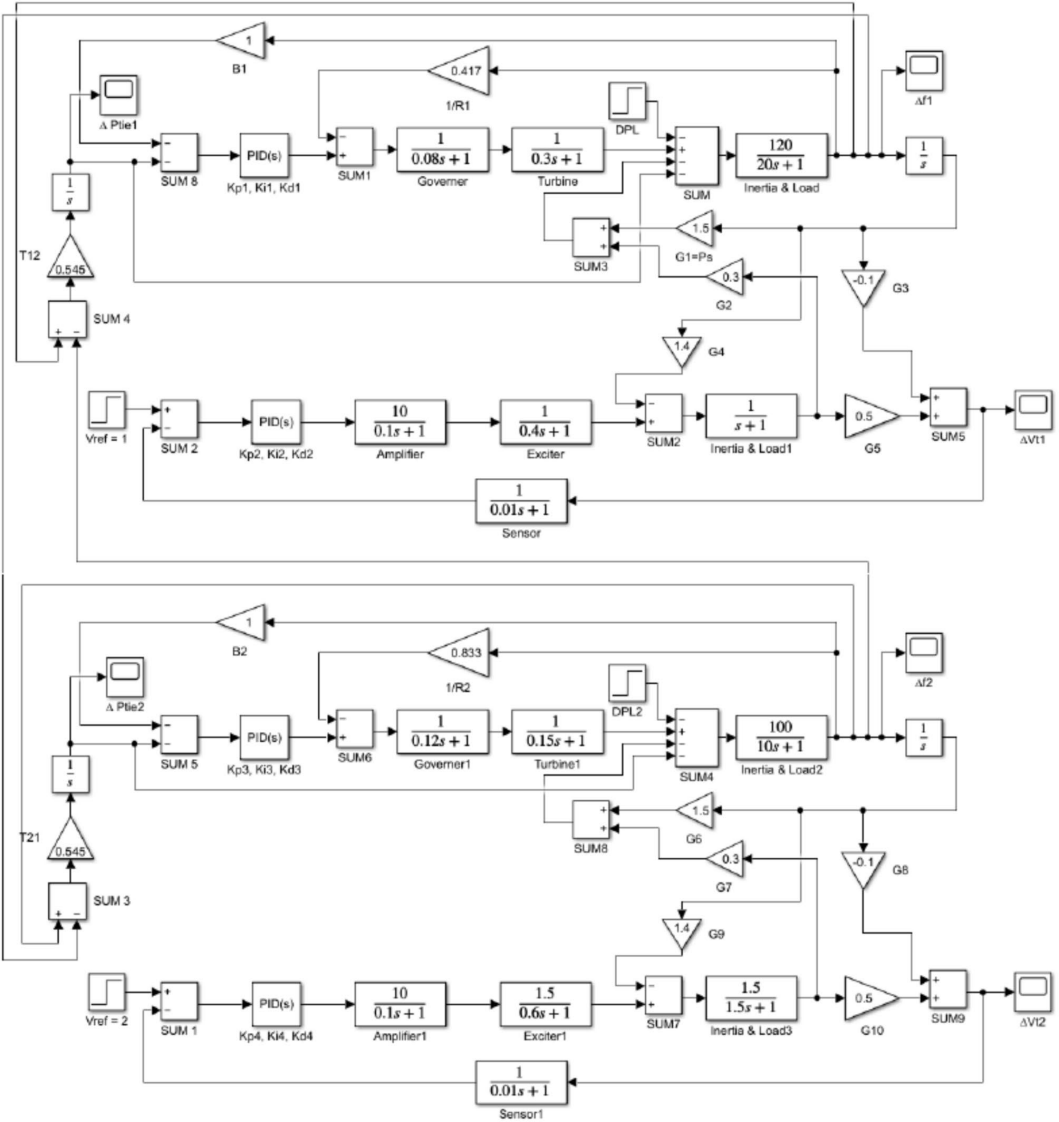
Two-area IPS with combined LFC − AVR.

The two-area IPS model under study is shown in Fig. 16. The terminal voltage was maintained at nominal value by stabilizing the generator fields, while the load frequency was regulated by controlling real power ^34^.

The proposed combined LFC and AVR system parameters^13^ for the two-area model are mentioned in Table 8, as well as the tuned parameters of the proposed EDO-PID controller, which improved the dynamic performance of the system much better than the NLTA-PID controller illustrated in Table 9.

**Table 8:** The values of system parameters (Area 1 and 2).

Area-1				Area-2			
B1	1	Ke1	1	B2	1	Ke2	1.5
R1	2.4	Te1	0.4	R2	1.2	Te2	0.6
KG1	1	Kg1	1	KG2	1	Kg2	1.5
TG1	0.08	Tg1	1	TG2	0.12	Tg2	1.5
Kt1	1	Ks1	1	Kt2	1	Ks2	1
Tt1	0.3	Ts1	0.01	Tt2	0.15	Ts2	0.01
∆ PD1	0.02	G1	1.5	∆ PD2	0.02	G6	1.5
Kp1	120	G2	0.3	Kp2	100	G7	0.3
Tp1	20	G3	0.1	Tp2	10	G8	0.1
Ka1	10	G4	1.4	Ka2	10	G9	1.4
Ta1	0.1	G5	0.5	Ta2	0.1	G10	0.5
T12	0.545			T21	0.545

**Table 9:** Optimal values of controller parameters.

Parameters	NLTA-PID^34^	EDO-PID
*Kp1*	1.995	3.6052
*Ki1*	1.943	0.4528
*Kd1*	1.079	2.2950
*Kp2*	1.994	1.2615
*Ki2*	1.295	0.7611
*Kd2*	1.107	0.4342
*Kp3*	1.956	3.1128
*Ki3*	1.919	0.7942
*Kd3*	0.655	1.0917
*Kp4*	1.283	2.0953
*Ki4*	0.586	1.2012
*Kd4*	0.819	0.7558

### Transient response

Tables 10 and 11 show the summary of LFC and AVR responses using NLTA-PID, and EDO-PID controller in a two-area IPS, respectively.

**Table 10:** Frequency deviation characteristics and PID tuning using EDO and NLTA (area-1) (area-2).

	Freq. (Area-1)				Freq. (Area-2)			
	Md (p.u)	tp (sec.)	ts (sec.) ± 5%	Ess (p.u)	Md (p.u)	tp (sec.)	ts (sec.) ± 5%	Ess (p.u)
EDO	− 0.1173	0	5.20	0	− 0.1121	0	5.617	0.0006
NLTA^34^	− 0.1298	0.00011	5.286	0	− 0.1146	0.0001	6.357	0.0002

**Table 11:** Voltage deviation characteristics and PID tuning using EDO and NLTA (area-1) (area-2).

	Voltage (Area-2)				Voltage (Area-2)				
	Mp (p.u)	tp (sec.)	ts (sec.) ± 2%	Ess (p.u)	Mp (p.u)		tp (sec.)	ts (sec.) ± 2%	Ess (p.u)
EDO	1.11	1.82	3.59	0.0003	1.135		1.52	3.42	0.0003
NLTA^34^	1.157	2.23	4.23	− 0.0004	1.151		2.62	5.25	0.0004

**Fig. 17:**
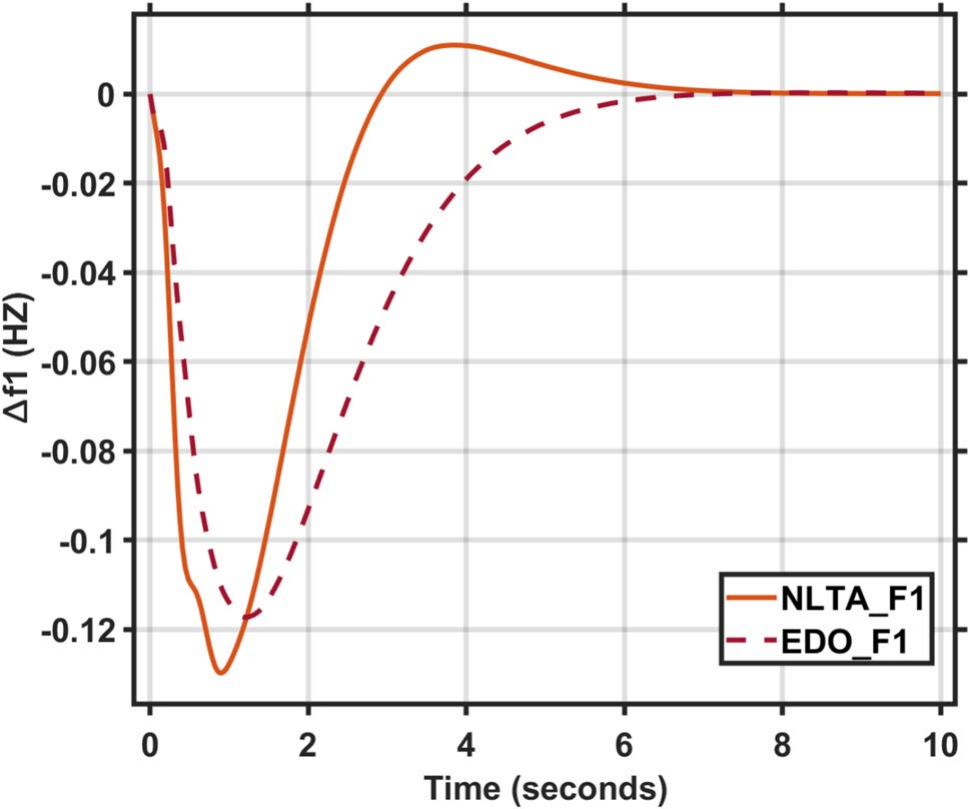
LFC response with PID NLTA and EDO based ∆f 1.

**Fig. 18:**
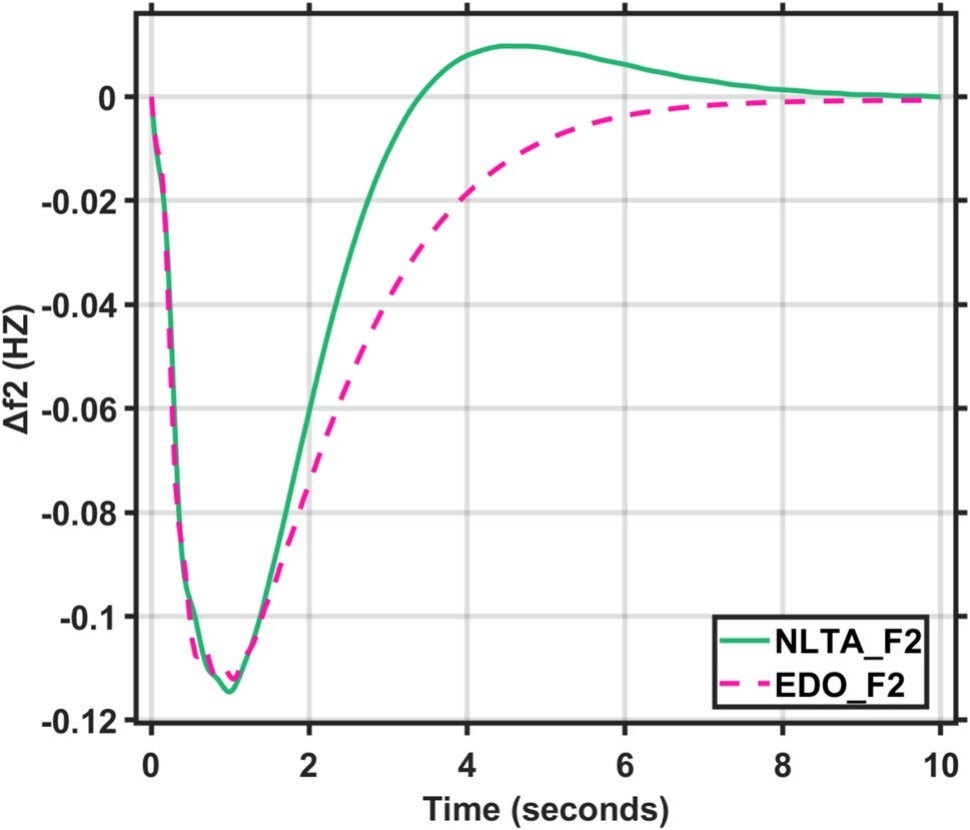
LFC response with PID NLTA and EDO based ∆f 2.

**Fig. 19:**
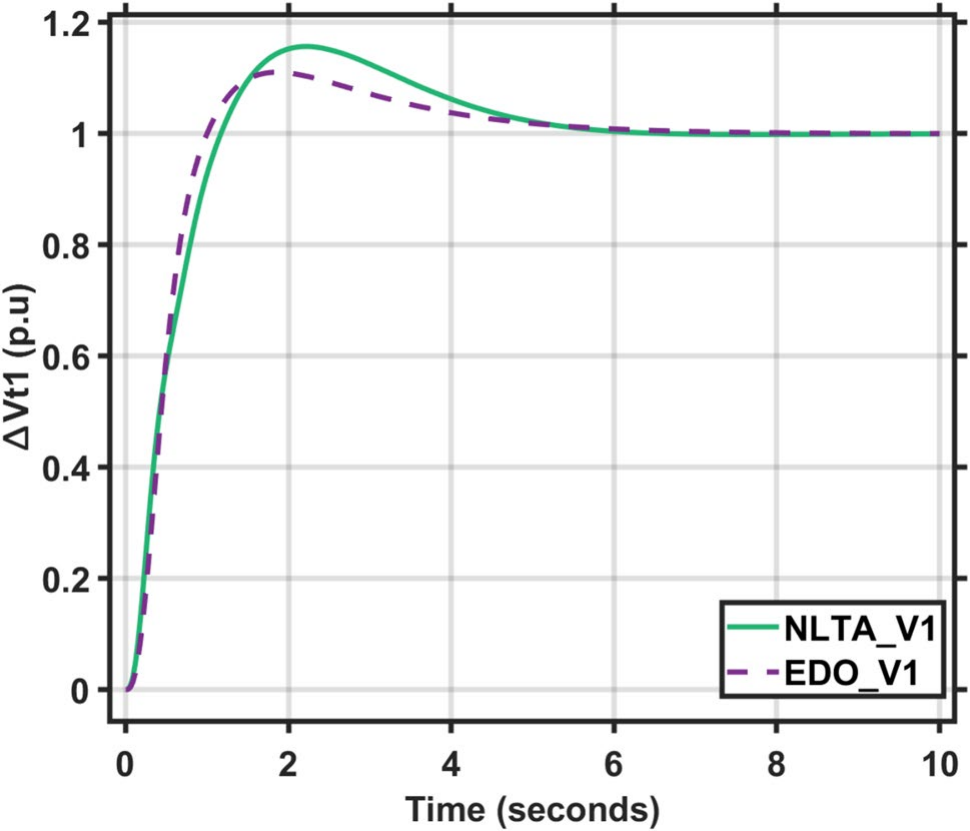
AVR response with PID NLTA and EDO based Vt1.

**Fig. 20:**
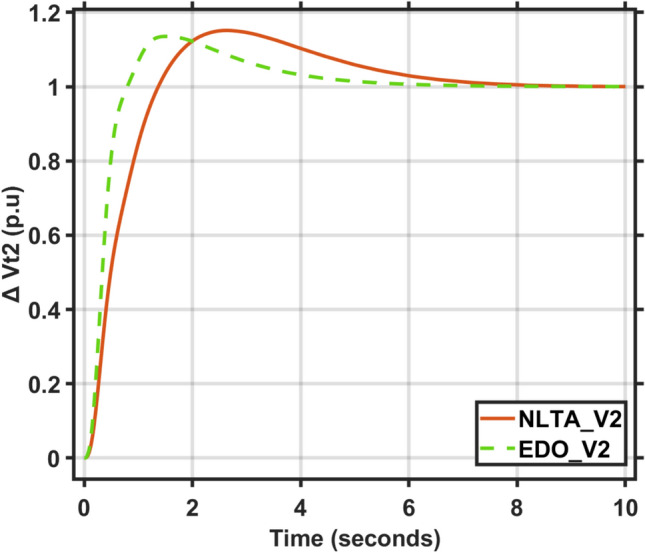
AVR response with PID NLTA and EDO based Vt2.

**Fig. 21:**
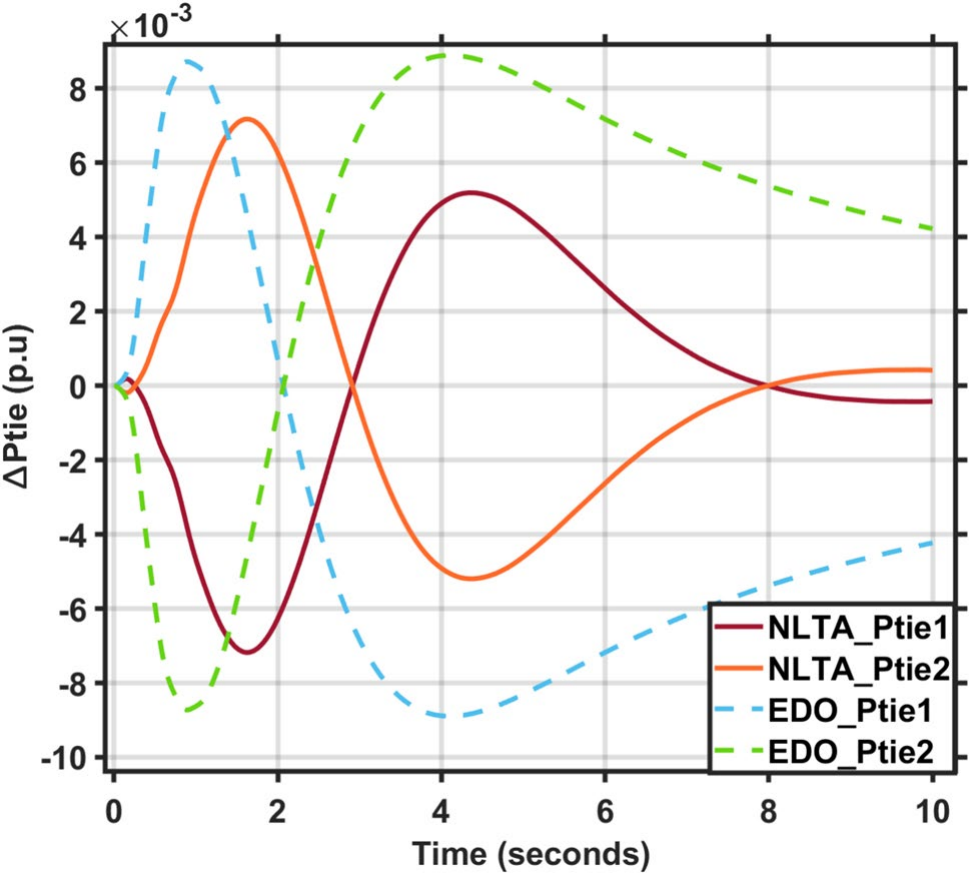
Tie line response for multi-area.

Figures 17 and 18 show the frequency deviation curves of area-1 and area-2 using NLTA-PID^15^ and EDO-PID control techniques in a two-area IPS. The proposed control schemes provided a very satisfactory frequency deviation response. For the area-1 LFC, the settling time of NLTA-PID^15^ was higher than the proposed schemes by 1.65%. NLTA-PID provided an undershoot of − 0.1298, whereas the proposed EDO-PID provided − 0.1173. It can be noticed that the proposed EDO-PID provided 10.7% of the better undershoot responses as compared to the NLTA-PID controller in area-1. For area-2, NLTA-PID provided a slow settling; it provided an undershoot of − 0.1146, whereas the proposed EDO-PID provided − 0.1121. It was verified that the proposed EDO-PID provided 2.23% better undershoot responses as compared to the NLTA-PID controller. The steady state (s–s) errors were almost zero with each proposed technique. For the area-1 LFC, the overshoot of NLTA-PID^5^ was higher than the proposed schemes. NLTA-PID provided an overshoot percentage of 0.00011, whereas the proposed EDO-PID provided zero. It can be noticed that the proposed EDO-PID provided better undershoot responses as compared to the NLTA-PID controller in area-1. For the area-2 LFC, the overshoot of NLTA-PID ^5^ was higher than the proposed schemes. NLTA-PID provided an overshoot percentage of 0.0001, whereas the proposed EDO-PID provided zero. It can be noticed that the proposed EDO-PID provided better undershoot responses as compared to the NLTA-PID controller in area-1. Figures 19 and 20 show the terminal voltage of area-1 and area-2 using the NLTA-PID and EDO-PID control techniques in a two-area IPS, respectively. Figure 21 shows the tie-line response. The proposed control schemes provided a very satisfactory transient response in both area-1 and area-2. It is identified that EDO-PID provided 4.23% and 1.4% overshoot in area-1 and area-2, respectively, and better settling time with the proposed technique. It can be observed that the proposed EDO-PID controller produced better steady state error than NLTA-PID.

### Robustness analysis

**Fig. 22:**
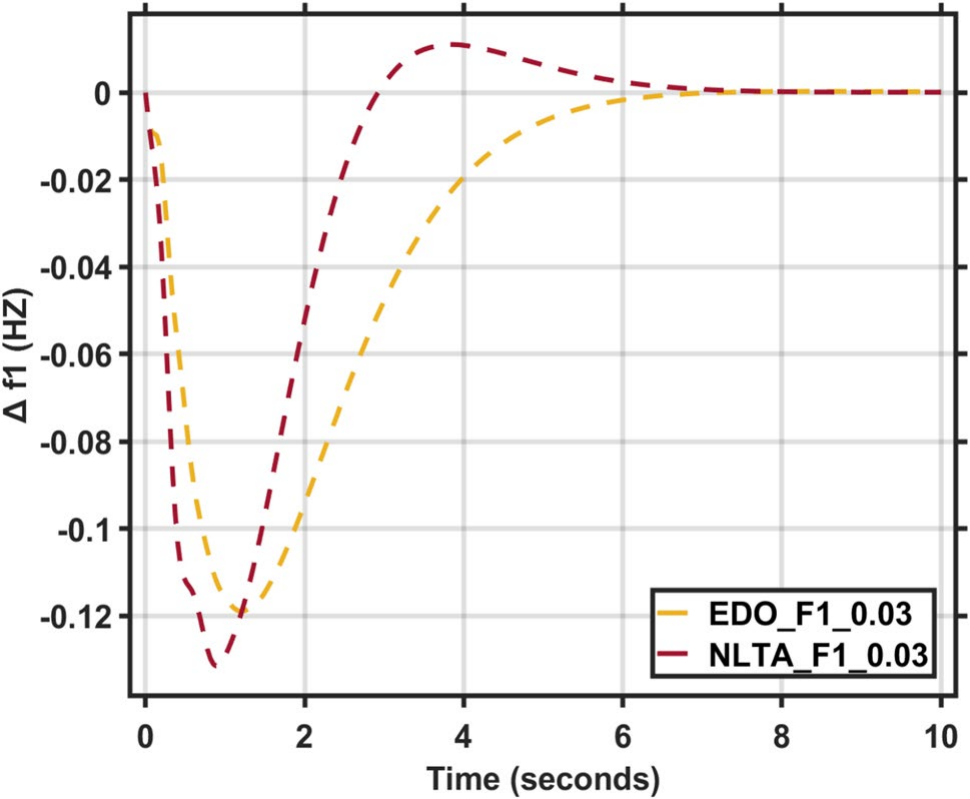
LFC response with PID NLTA and EDO based for load deviations (∆Pl1,2 = 0.03) and frequency deviation (∆f1).

**Fig. 23:**
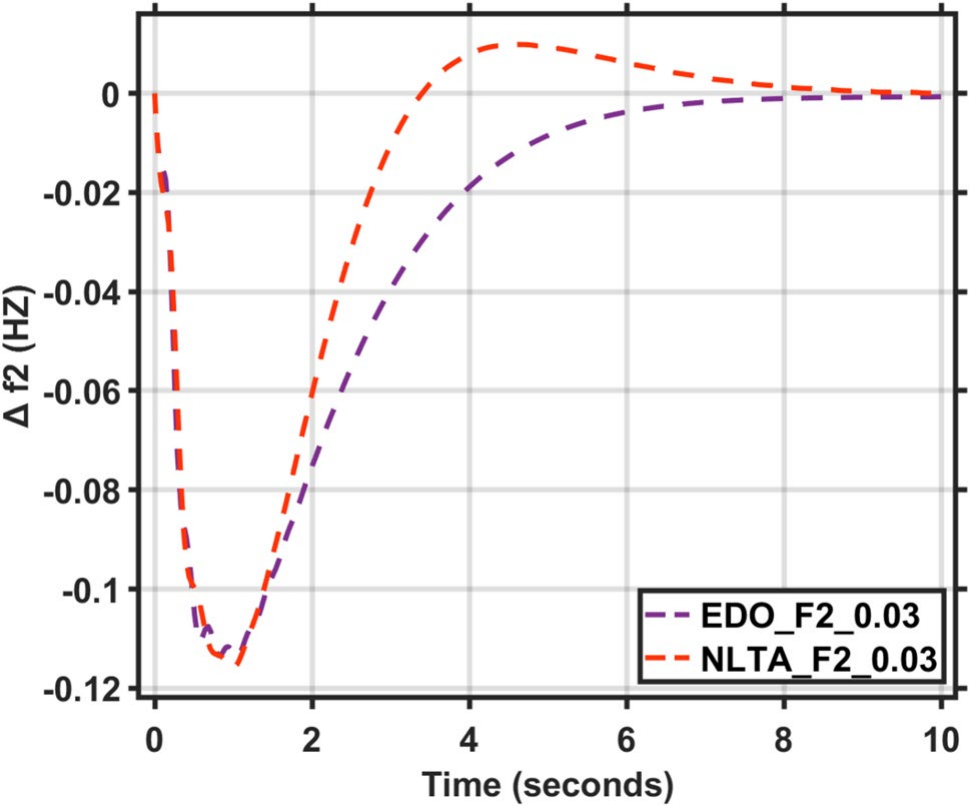
LFC response with PID NLTA and EDO based for load deviations (∆Pl1,2 = 0.03) and frequency deviation (∆f2).

**Fig. 24:**
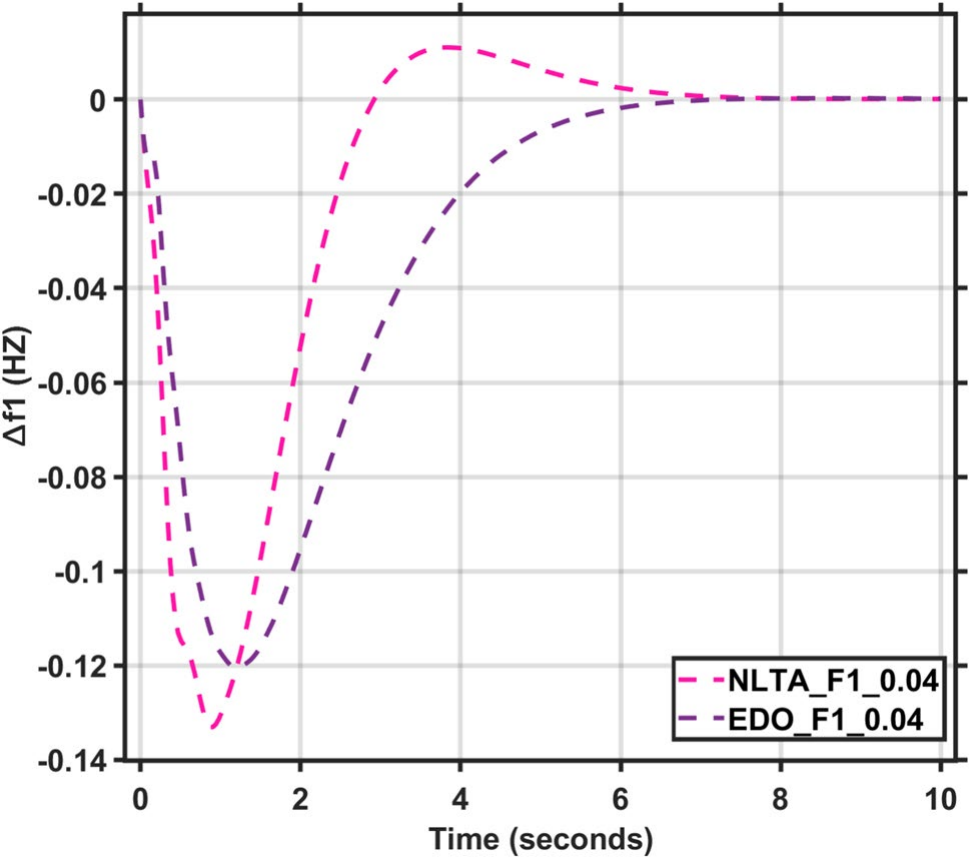
LFC response with PID NLTA and EDO based on load deviations (∆Pl1,2 = 0.04) and frequency deviation (∆f1).

**Fig. 25:**
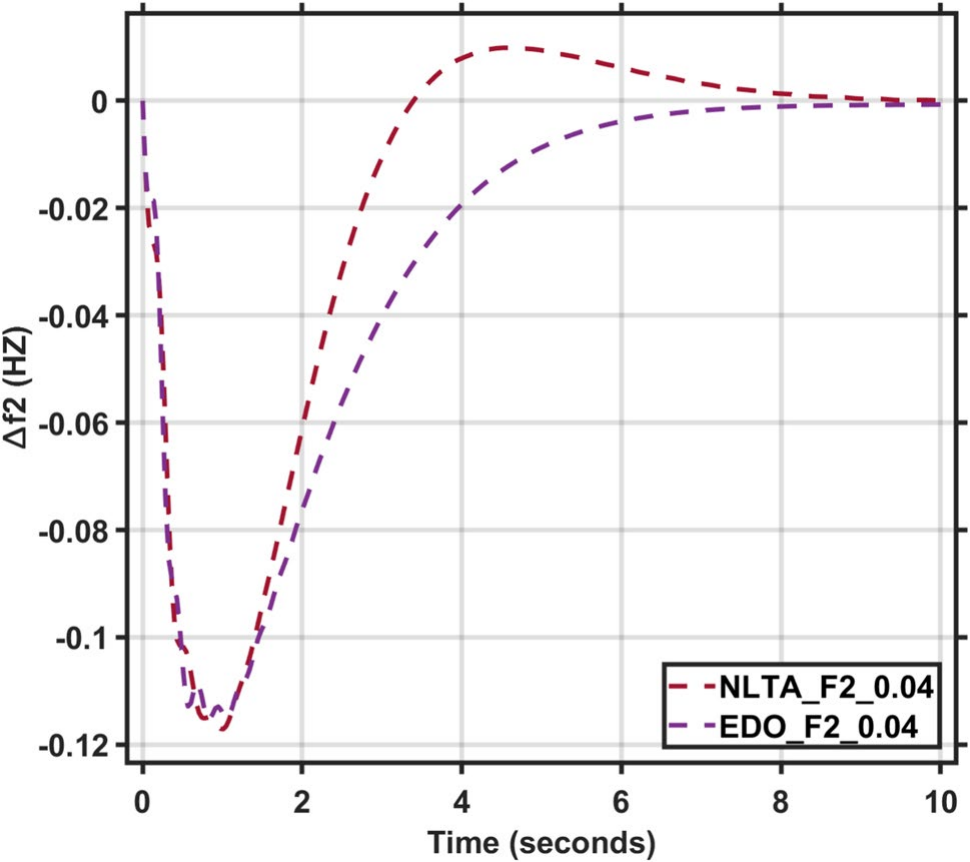
LFC response with PID NLTA and EDO based on load deviations (∆Pl1,2 = 0.04) and frequency deviation (∆f2).

In this section, the reliability and effectiveness of the proposed method is proved through applying load deviations represented by different values of the step function (∆Pl) in both areas, ranging from zero to 0.02, 0.03, and 0.04, the dynamic response of the combined LFC and AVR system was evaluated under the tuned proposed EDO-PID controller compared to the PID-NLTA. The frequency deviation characteristics and PID tuning using EDO and NLTA are summarized in Table 11 and the system response under deviation in load is shown in Figs. 22, 23, 24 and 25. The results show that the LFC response with the EDO-PID controller exhibited better undershoots by 10.51%, 1.85%, 10.46%, and 1.91% for area-1 and area-2 compared to PID-NLTA. Furthermore, the EDO-PID controller achieved zero overshoot, whereas the PID-NLTA showed overshoots of 0.01106 p.u., 0.0099 p.u., 0.01105 p.u., and 0.0099 p.u. respectively. The EDO-PID controller also provided a faster dynamic response and improved steady-state error.

**Fig. 26:**
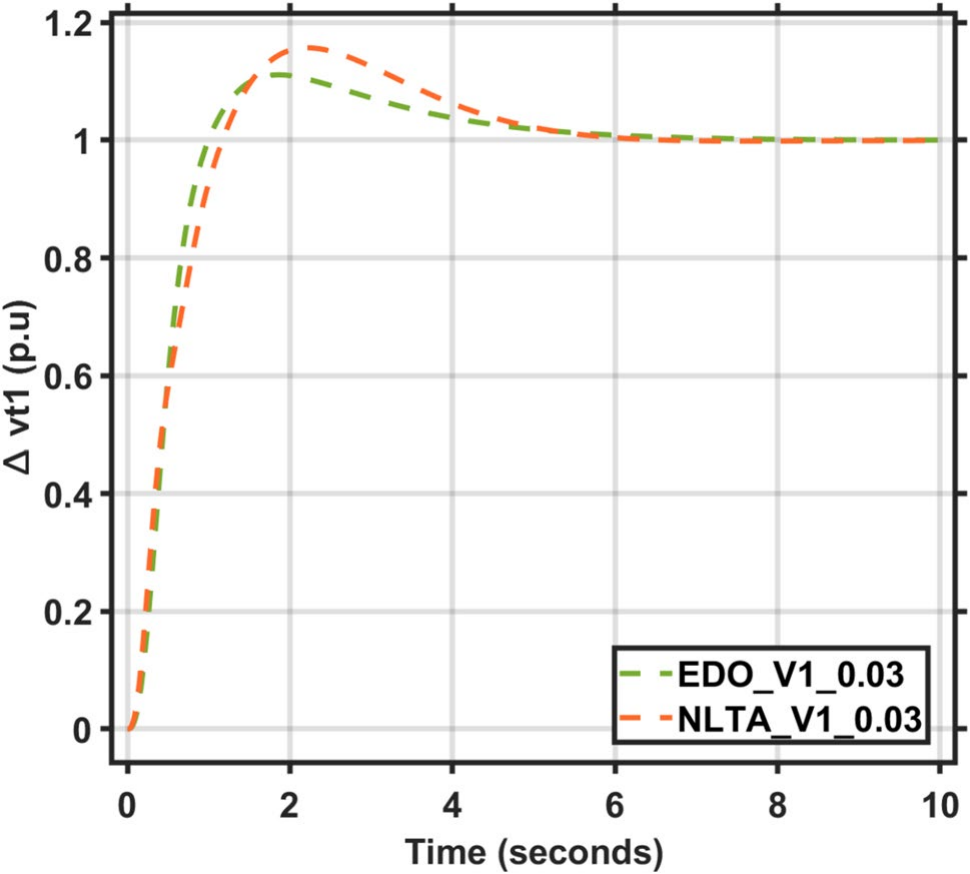
AVR response with PID NLTA and EDO based on load deviations (∆Pl1,2 = 0.03) and voltage deviation (∆v1).

**Fig. 27:**
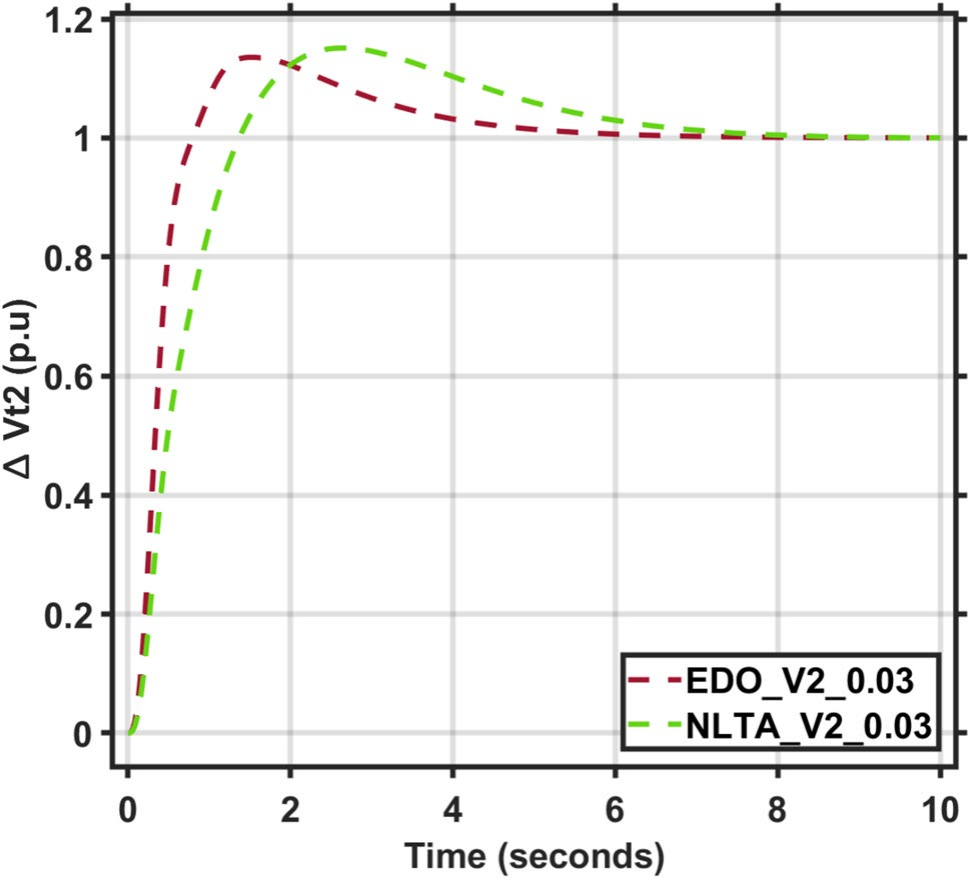
AVR response with PID NLTA and EDO based on load deviations (∆Pl1,2 = 0.03) and voltage deviation (∆v2).

**Fig. 28:**
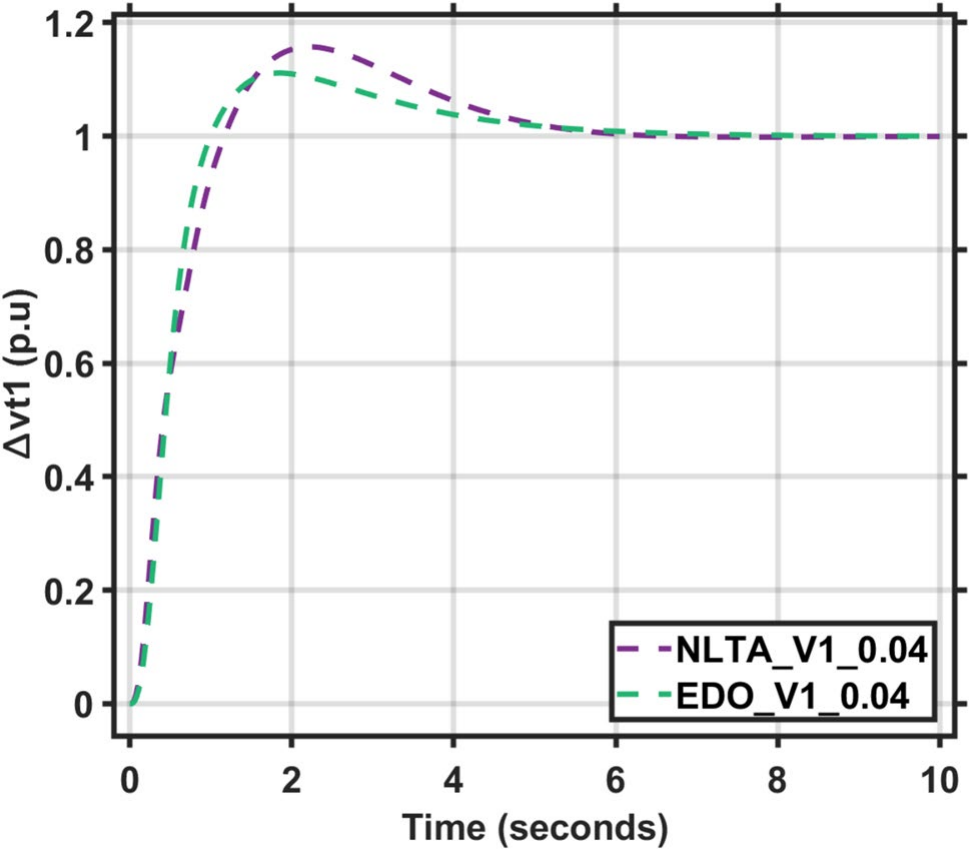
AVR response with PID NLTA and EDO based on load deviations (∆Pl1,2 = 0.04) and voltage deviation (∆v1).

**Fig. 29:**
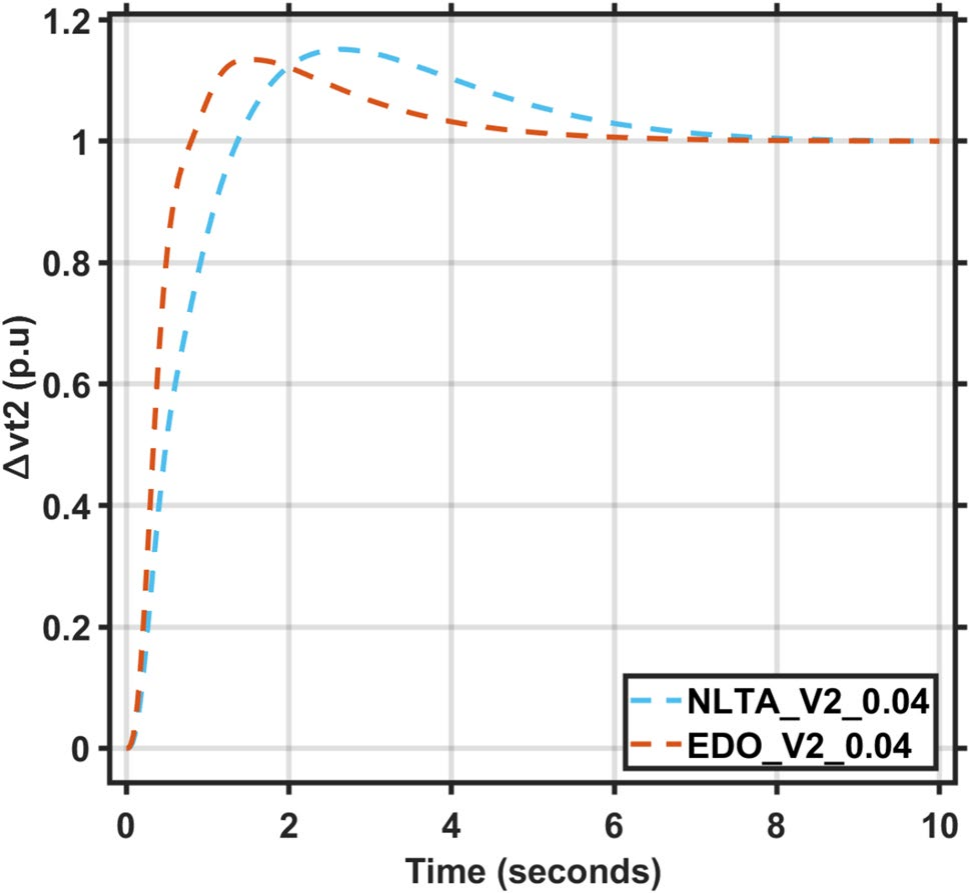
AVR response with PID NLTA and EDO based on load deviations (∆Pl1,2 = 0.04) and voltage deviation (∆v2).

The voltage deviation characteristics and PID gains using EDO and NLTA, as illustrated in Table 12 and Figs. 26, 27, 28 and 29, reveal that the AVR response with the EDO-PID controller exhibited 4.14%, 1.37%, 4.14%, and 1.49% better overshoots for area-1 and area-2 compared to the PID-NLTA. Additionally, the settling time improved by 4.29%, 41.3%, 3.67%, and 40.69%, respectively, when compared to the PID-NLTA. The EDO-PID controller also provided a faster dynamic response and better steady-state error (Table 13).

**Table 12:** Frequency deviation characteristics and PID tuning using EDO and NLTA for area-1 and area-2, with load deviations (∆Pl1,2) set to 0.03 and 0.04.

	Freq. (Area-1)				Freq. (Area-2)			
	Md (p.u)	tp (sec.)	ts (sec.) ± 5%	Ess (p.u)	Md (p.u)	tp (sec.)	ts (sec.) ± 5%	Ess (p.u)
EDO (0.03)	− 0.1189	0	6.870	− 0.0002	-0.1137	0	9.37	0.0007
NLTA (0.03)	− 0.1314	0.01106	9.272	− 0.0001	-0.1158	0.0099	9.74	− 0.0001
EDO (0.04)	− 0.1205	0	9.66	− 0.0002	0.1149	0	9.36	0.0007
NLTA (0.04)	− 0.1331	0.01105	9.56	− 0.0001	-0.1171	0.0099	9.33	− 0.0003

**Table 13:** Voltage deviation characteristics and PID tuning using EDO and NLTA for area-1 and area-2 with load deviations (∆Pl1,2 = 0.03 and 0.04).

	Voltage.(Area-1)				Voltage.(Area-2)				
	Mp (p.u)	tp (sec.)	ts (sec.) ± 2%	Ess (p.u)	Mp (p.u)		tp (sec.)	ts (sec.) ± 2%	Ess (p.u)
EDO (0.03)	1.111	1.852	4.9	− 3.86 × 10^–4^	1.1357		1.516	4.6	− 2.672 × 10^–4^
NLTA (0.03)	1.157	2.189	5.11	4.72 × 10^–4^	1.1513		2.649	6.5	− 1.988 × 10^–4^
EDO (0.04)	1.111	1.808	4.9	− 1.9 × 10^–4^	1.1346		1.473	4.62	− 2.998 × 10^–4^
NLTA (0.04)	1.157	2.238	5.08	4.8 × 10^–4^	1.1515		2.609	6.5	− 1.461 × 10^–4^

## Conclusion and future work

The study on the Exponential Distribution Optimization (EDO)-based PID controller demonstrated its effectiveness in enhancing the dynamic response of interconnected power systems (IPS). In a single-area IPS, the EDO-PID scheme outperformed PID controller FA based, achieving a 16.52% improvement in load frequency control (LFC) undershoots, a 2.86% better LFC steady-state error, a 1.31% reduction in automatic voltage regulation (AVR) overshoots, and a 33.3% improvement in AVR steady-state error. For a two-area IPS, it surpassed the PID controller NLTA based, reducing LFC undershoots by 10.7% in Area-1 and 2.23% in Area-2, improving AVR overshoots by 4.23% in Area-1 and 1.41% in Area-2, and delivering faster settling times for LFC (1.65% in Area-1, 13.2% in Area-2) and AVR rise times (22.5% in Area-1, 17.8% in Area-2), alongside significantly quicker AVR settling times (72.4% in Area-2, 52.5% in Area-1). Overall, the EDO-PID scheme achieved zero steady-state error, effectively managed frequency overshoot and transient oscillations, and reduced transient oscillations, making it highly suitable for real-world multi-area IPS applications, with potential for further improvement through integrating multi-source energy and energy storage devices.

In the future, researchers should prioritize several key areas to advance load frequency control (LFC) in power systems. Applying proposed techniques to multi-area systems can improve stability, enhance transient responses, and reduce steady-state errors. Integrating adaptive controllers could provide superior stability and dynamic performance compared to traditional PID controllers. Additionally, leveraging advanced optimization techniques may enable more precise control strategies. Addressing grid variability by incorporating disturbances from renewable energy sources, such as wind and solar, is essential for modern power systems. Finally, modeling battery charge and discharge behaviors could harness energy storage systems to bolster frequency regulation. Together, these efforts aim to develop more robust, adaptive, and efficient power systems.

## Data Availability

The datasets used and/or analyzed during the current study are available from the corresponding author on reasonable request.

## Abbreviations

PID: Proportional integral derivative
LFC: Load frequency control
AVR: Automatic voltage regulator
EDO: Exponential distribution optimization
ITSE: Integral time square error
ITAE: Integral time absolute error
IPS: Interconnected power system
*Kp*: Proportional gain
*Ki*: Integral gain
*Kd*: Derivative gain
*Ri*: Speed regulation
*T12, T21*: Tie-line synchronizing time constants
*KA*: Amplifier gain
*KE*: Exciter gain
*KG*: Generator gain
*KS*: Sensor gain
*τA*: Amplifier time constant
*τE*: Exciter time constant
*τG*: Generator time constant
*τS*: Sensor time constant
*Vref*: Reference voltage
*∆f*: Frequency deviation
*∆PD*: Load deviation
*∆**Ptie*: Tie-line power deviation
